# Constitutive patterns of gene expression regulated by RNA-binding proteins

**DOI:** 10.1186/gb-2014-15-1-r13

**Published:** 2014-01-02

**Authors:** Davide Cirillo, Domenica Marchese, Federico Agostini, Carmen Maria Livi, Teresa Botta-Orfila, Gian Gaetano Tartaglia

**Affiliations:** 1 Gene Function and Evolution, Centre for Genomic Regulation (CRG), Dr Aiguader 88, Barcelona 08003, Spain; 2 Universitat Pompeu Fabra (UPF), Barcelona 08003, Spain

## Abstract

**Background:**

RNA-binding proteins regulate a number of cellular processes, including synthesis, folding, translocation, assembly and clearance of RNAs. Recent studies have reported that an unexpectedly large number of proteins are able to interact with RNA, but the partners of many RNA-binding proteins are still uncharacterized.

**Results:**

We combined prediction of ribonucleoprotein interactions, based on catRAPID calculations, with analysis of protein and RNA expression profiles from human tissues. We found strong interaction propensities for both positively and negatively correlated expression patterns. Our integration of *in silico* and *ex vivo* data unraveled two major types of protein–RNA interactions, with positively correlated patterns related to cell cycle control and negatively correlated patterns related to survival, growth and differentiation. To facilitate the investigation of protein–RNA interactions and expression networks, we developed the catRAPID express web server.

**Conclusions:**

Our analysis sheds light on the role of RNA-binding proteins in regulating proliferation and differentiation processes, and we provide a data exploration tool to aid future experimental studies.

## Background

With the advent of high-throughput proteomic and transcriptomic methods, genome-wide data are giving previously unprecedented views of entire collections of gene products and their regulation. Recently, approaches based on nucleotide-enhanced UV cross-linking and oligo(dT) purification have shown that a number of proteins are able to bind to RNA [1,2].

RNA-binding proteins (RBPs) are key regulators of post-transcriptional events [3] and influence gene expression by acting at various steps in RNA metabolism, including stabilization, processing, storing, transport and translation. RBP-mediated events have been described using recognition and regulatory elements in RNA sequences [4,5] as well as expression profiles [6] that are tissue specific and conserved across species [7-9]. Although heterogeneity in gene regulation is responsible for phenotypic variation and evolution [10], very little is known about constitutive expression patterns controlled by RBPs [11,12], which are the subject of this work.

Data from recent transcriptomic and proteomic studies [13,14] are becoming attractive for studying mechanisms of gene regulation [15,16]. Despite the increasing amount of genomic data, the development of computational methods for integrating, interpreting and understanding molecular networks remains challenging [17,18]. Here we combine our predictions of protein–RNA interactions, based on catRAPID calculations [19,20], with the information obtained from expression data to investigate constitutive regulatory mechanisms. The catRAPID approach has been previously employed to predict protein associations with non-coding RNAs [21,22] as well as ribonucleoprotein interactions linked to neurodegenerative diseases [23,24]. Our theoretical framework has been used to unravel self-regulatory pathways controlling gene expression [25]. The catRAPID omics algorithm, validated using photoactivatable-ribonucleoside-enhanced cross-linking and immunoprecipitation (PAR-CLIP) data, has been recently developed to predict protein–RNA associations at the transcriptomic and proteomic levels [26].

Using comprehensive and manually annotated databases of expression profiles in human tissues, at both protein and RNA levels, we investigated the correlation between RBP activity and regulation. The link between interaction propensity and expression levels was exploited to reveal the fine-tuned functional sub-networks responsible for regulatory control. To explore the results further, we developed the catRAPID express web server [27].

## Results

In this study, we focused on the mRNA interactomes of RBPs detected through nucleotide-enhanced UV cross-linking and oligo(dT) purification approaches [1,2]. Exploiting gene ontology (GO) annotations [28] for protein-coding genes, we systematically analyzed protein–RNA interactions and expression data for human tissues.

At present, few studies have investigated how altering protein expression affects the abundance of RNA targets. Interrogating the Gene Expression Omnibus (GEO) [29] and ArrayExpress databases [30], we found two human proteins, ELAV-like protein 1 (or human antigen R, HuR) [31] and Protein lin-28 homolog B (LIN28B) [32,33], whose knock-down has been shown to alter the expression of target genes identified by PAR-CLIP (see Materials and methods).

Our predictions, made using the catRAPID algorithm [26], identified experimentally validated interactions with high significance (HuR: *P* = 10^-8^; LIN28B: *P* = 10^-3^; Fisher’s exact test; see Materials and methods). The interactions were effectively discriminated from non-interacting pairs using score distributions (LIN28B: *P* = 10^-4^; HuR: *P* = 10^-16^; Student’s t-test; see Materials and methods). Hence, catRAPID is very good at predicting physical interactions between a protein and RNA partners (other statistical tests are given in Materials and methods and Additional file 1).

**Figure 1:**
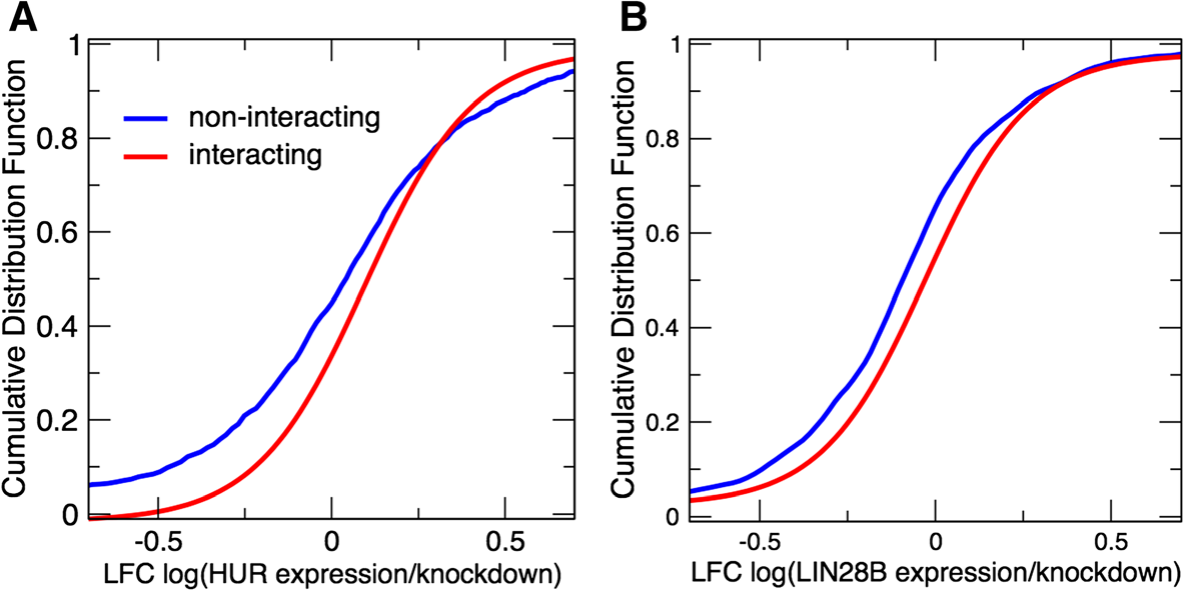
**Relation between protein and RNA regulation. (A)** HuR interactome: our predictions, made using catRAPID [26], indicate that expression levels of RNA targets change upon HuR knock-down (log-fold changes, LFC), in agreement with experimental evidence [31] (*P* < 10^-5^; Kolmogorov–Smirnov test). **(B)** LIN28B interactome: RNA targets are downregulated upon LIN28B knock-down (LFC), as reported in a previous study [33] (*P* < 10^-2^; Kolmogorov–Smirnov test). In this analysis, the prediction of the interactions was highly significant (HuR: *P* < 10^-8^; LIN28B: *P* < 10^-3^; Fisher’s exact test). Our results indicate that changes in protein expression influence the abundance of RNA targets to a significant extent. HuR, human antigen R; LFC, log-fold change; LIN28B, lin-28 homolog B.

To understand the regulation of HuR and LIN28B targets better, we studied the relation between interaction propensities and expression levels. We found that the expression of predicted HuR targets is altered (log-fold change, LFC) when HuR is knocked down (*P* < 10^-5^; Kolmogorov–Smirnov test; Figure 1A), which is in agreement with experimental data [31]. Similarly, predicted LIN28B targets are downregulated upon protein depletion (*P* < 10^-2^; Kolmogorov–Smirnov test; Figure 1B), as shown in a previous study [33]. Moreover, we compared the top 1% of predicted associations with the top 1% of experimental interactions and found the same enrichments for transcripts changing in expression levels upon protein depletion. Specifically, 62% of HuR experimental interactions and 63% of HuR predicted associations had LFC > 0. Similarly for LIN28B, 57% of experimental interactions and 56% of predicted associations had LFC > 0.

These HuR and LIN28B examples indicate that changes in protein expression influence the abundance of RNA targets, suggesting that a large-scale analysis of co-expression and interaction propensities could improve understanding of RBP-mediated regulatory mechanisms.

### RNA-binding protein–mRNA interactions and relative expression profiles

**Figure 2:**
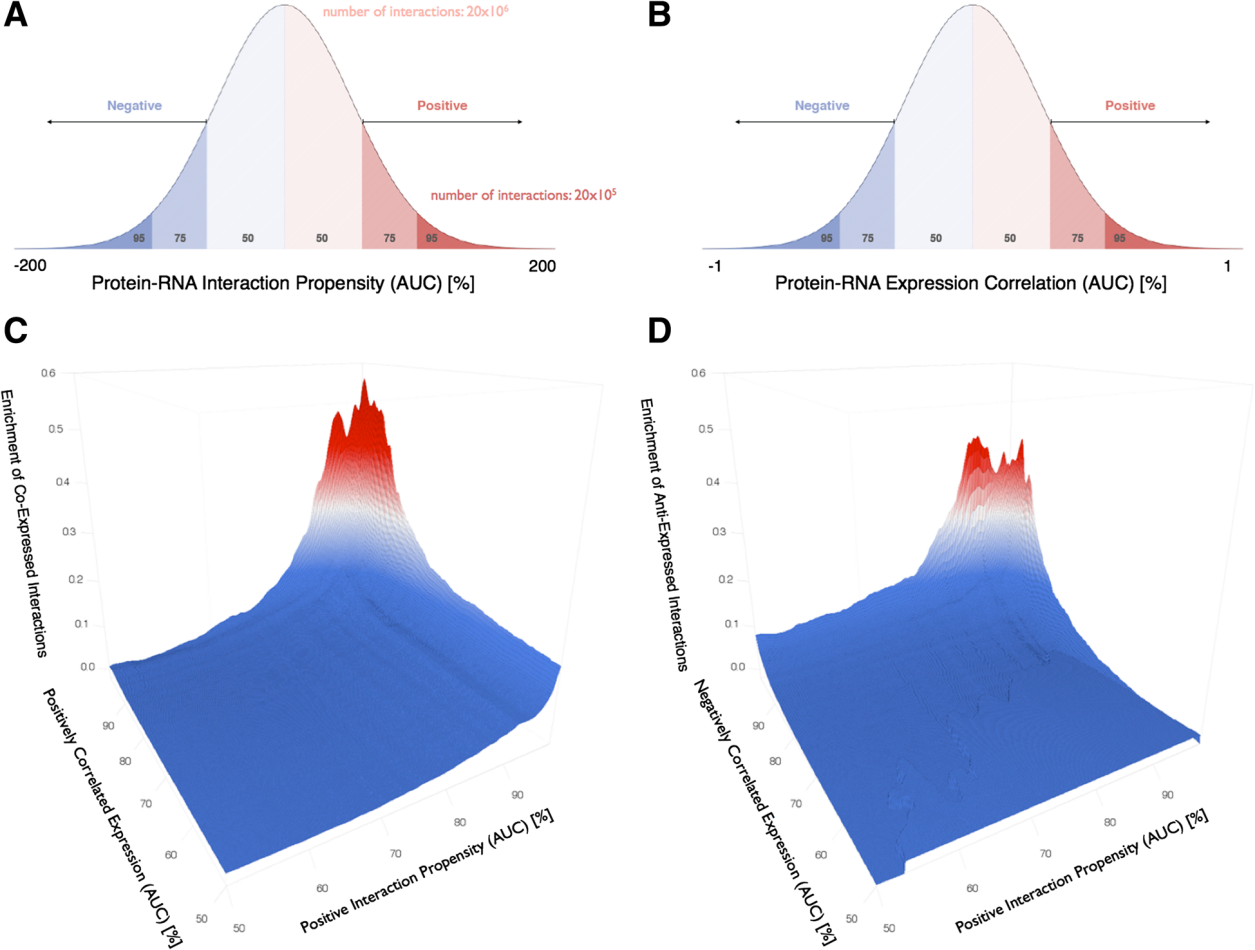
**Protein–RNA interaction and expression. (A)** In this analysis, we compared interacting and non-interacting protein–RNA pairs at different interaction propensity scores. Areas under the curve (AUCs), expressed as percentages, were used to select the same number of interacting and non-interacting protein–RNA pairs. **(B)** The same procedure was used to investigate positively and negatively correlated protein–RNA expression at different thresholds. **(C)** With respect to non-interacting protein–RNA pairs, the predicted associations had enriched positively correlated expression (that is, co-expression; see Materials and methods). **(D)** Compared to non-interacting protein–RNA pairs, the predicted associations had enriched negatively correlated expression (that is, anti-expression; see Materials and methods). Non-correlated protein–RNA expression did not show any similar trend (Additional file 1). AUC, area under the curve.

Our predictions indicate that interacting molecules have both more correlated and anti-correlated expression patterns (see Materials and methods and Figure 2). By contrast, non-correlated expression is not associated with any enrichment in interaction propensity (Additional file 2: Figure S1A). We observed the same results using immunohistochemistry [34] and RNA sequencing data [6] to estimate protein abundances (Additional file 2: Figures S1B and S2; see Materials and methods). This finding is truly remarkable. Direct proportionality between protein and mRNA expression levels has been observed in bacteria and fungi [13,14] but post-transcriptional modification is known to influence the overall abundance of the protein product in higher eukaryotes [35]. Since immunohistochemistry only provides a qualitative estimate of the amount of protein (see Materials and methods) and the analysis is restricted to 612 proteins, we used RNA sequencing for our predictions (1,156 RBPs).

The enrichment shown in Figure 2 suggests that a good relation exists between interaction and expression of protein–RNA molecules, which should have co-evolved to be either co-expressed or anti-expressed to exert a regulatory function (Figure 2C,D).

### Conservation of expression pattern for functionally related genes

We classified protein–RNA associations into four categories: interacting and co-expressed (IC), interacting and anti-expressed (IA), non-interacting and co-expressed (NIC) and non-interacting and anti-expressed (NIA). We applied conditional tests on each subset to detect significantly over-represented gene ontology (GO) terms (see Materials and methods and Additional file 3: Table S1).

For high interaction propensities, transcripts in the IC subset have more processes associated with *cell cycle control*, in particular the negative regulation of proliferation (Discussion; Additional file 3: Table S1).

Transcripts interacting with anti-expressed proteins (IA subset) are involved in *survival, growth and differentiation* processes and have more regulative functions at the DNA level (Discussion; Additional file 3: Table S1).

No clear functional assignments and/or insufficiently populated GO terms were found for transcripts in non-interacting protein–RNA pairs (NIC and NIA subsets).

### Intrinsic disorder and RNA-binding protein interaction propensity

**Figure 3:**
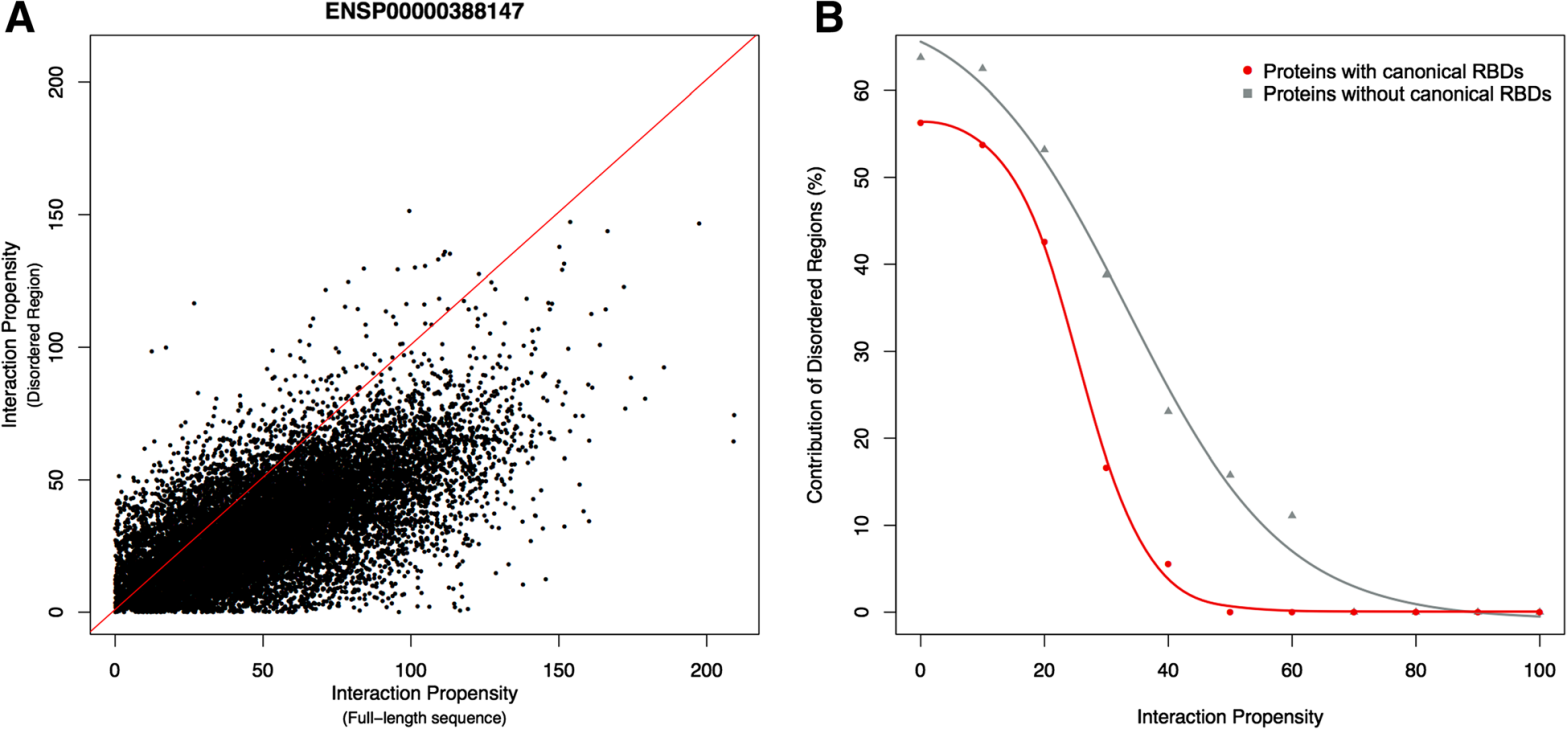
**RNA-binding ability and structural disorder. (A)** For each protein, we calculated RNA interactions with full-length sequences as well as structurally disordered regions [1,36]. When the interaction propensity score of a disordered region exceeds that of the full-length protein (points above the red line), disorder is considered to promote interaction with RNA molecules. **(B)** For 66% of the proteins (137 entries), disorder contributes at low interaction propensities, while full-length protein sequences dominate at high interaction propensities (Mann–Whitney U test). Overall, from low to high interaction propensities, the contribution of disorder decreases progressively with respect to that of the full-length protein (red and grey lines), in agreement with a previous analysis [25]. The role of disorder is more relevant in proteins lacking canonical RNA-binding domains (grey line), indicating that unstructured regions might have direct involvement in contacting RNA. Interaction propensities are averaged per protein. RBD, RNA-binding domain.

Recent findings suggest that RBPs have more structurally disordered regions [1]. To investigate the relation between disorder and RNA-binding ability, we used the IUPred algorithm [36]. For each protein, we extracted structurally disordered regions (IUPred score > 0.4 [1]) and calculated the interaction propensities with human transcripts. We considered both canonical RBPs (that is, containing RNA-binding domains) and putative RBPs (that is, lacking RNA-binding domains) [1]. With respect to the RNA-binding ability of full-length sequences, the contribution of disorder is higher at low interaction propensity scores and becomes negligible at high interaction propensities (see Materials and methods and Figure 3A). Nevertheless, the role of structural disorder is more pronounced in proteins lacking canonical RNA-binding domains, indicating that unfolded regions might be able to promote interactions with RNA (Figure 3B).

In a previous study we observed that catRAPID scores correlate with chemical affinities [21], which suggests that the interaction propensity can be used to estimate the strength of association [21,26]. Hence, our results indicate that structural disorder might contribute to low-affinity interactions with RNA (Figure 3A,B), which is in agreement with what has been observed for protein–protein associations [37,38]. As a matter of fact, it has been reported that disorder regions are able to promote promiscuous and non-specific interactions [39].

## Discussion

Because they are associated with transcriptional control of gene expression, RBPs play fundamental roles in health and disease. Indeed, by binding to their target mRNAs, RBPs can influence protein production at different levels (transcription, translation and protein/mRNA degradation). Protein–RNA complexes are very dynamic and can undergo extensive remodeling. Thus, they can control the spatiotemporal regulation of target gene expression and the overall switching *on and off* of the distinct sets of genes involved in biological processes such as cell cycle progression, cell differentiation, cell response to metabolic *stimuli* and stress conditions, organ morphogenesis and embryonic development.

### Co-expression and interaction propensity are features of cell cycle control

**Figure 4:**
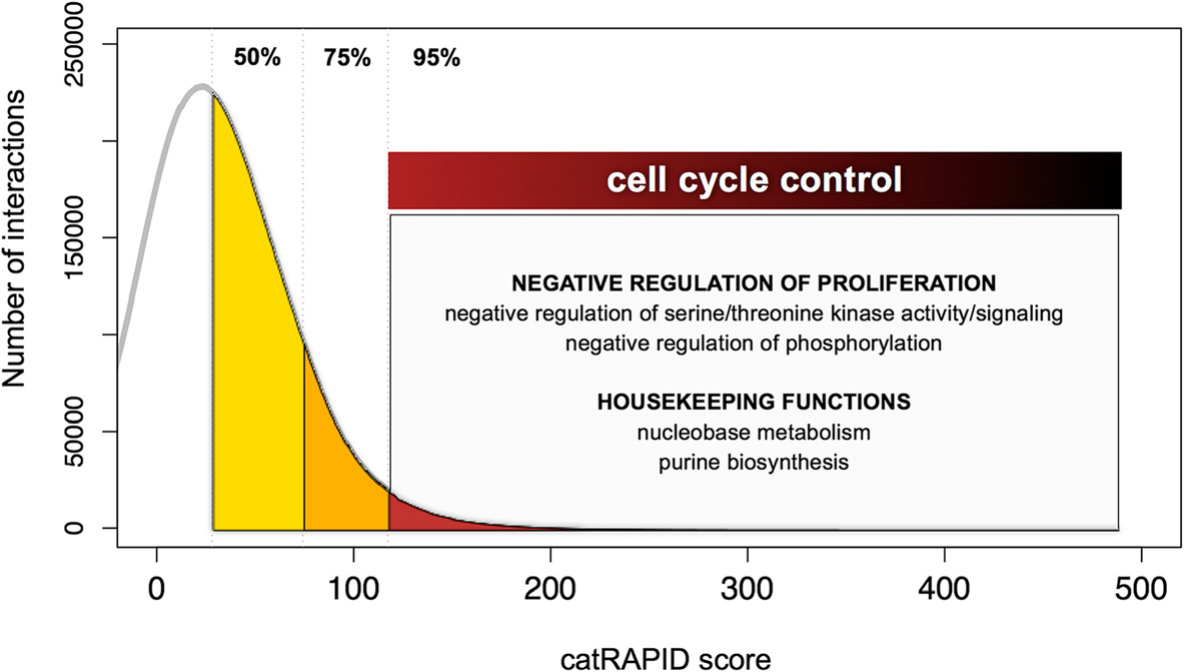
**GO enrichment for interacting mRNA–RBP pairs correlated in expression (IC subset).** Using the catRAPID score distribution, we counted mRNA GO enrichment associated with different areas under the curve (see Materials and methods). The color gradient (yellow to red) indicates the AUC values (number of interactions: 20,702,804 for AUC > 50%, 10,351,402 for AUC > 75%, 2,070,280 for AUC > 95%). We found that cell cycle processes have more highly interacting mRNA–RBP pairs (AUC > 95%) that are correlated in expression. AUC, area under the curve; GO, gene ontology; IC, interacting and co-expressed; RBP, RNA-binding protein.

At high interaction propensities (AUC > 95%; see Materials and methods), the IC subset has more GO terms linked to cell cycle control and housekeeping functions such as nucleobase metabolism and purine biosynthesis (Figure 4 and Additional file 3: Table S1). In particular, mRNAs interacting with co-expressed RBPs code for negative regulators of cell proliferation and migration (translation, signaling and metabolite utilization). We found a number of tumor suppressors in the IC subset (AHRR, BAX, BRMS1, CDKN1A, CDKN2A, CTBP1, DAB2IP, DKK3, FLCN, FOXP1, GADD45G, GALR1, GTPBP4, HIC1, IGFBP3, IRF8, KLF4, MEN1, MLH1, NF2, NR0B2, PARK2, PAWR, PAX4, PAX5, PCGF2, PHB, PML, PPP1R1B, PPP2R4, PTPRJ, PYCARD, RHOA, SIRT2, TFAP2A, TNFAIP3, TRIM24, TSC2, TSG101, UCHL1). Interestingly, 90% of IC genes annotated with more functional categories (381 out of 422) are listed in the gene index of the National Institutes of Health’s Cancer Genome Anatomy Project [40]. Terms associated with inhibition of cellular pathways (especially the negative regulation of phosphorylation and regulation of protein serine/threonine kinase activity) are also more prevalent in the IC subset when immunochemistry data are used.

As mutations altering tumor suppression lead to aberrant proliferative events, we speculate that downregulation of specific genes is a mechanism for preventing indiscriminate cellular growth. In agreement with this hypothesis, it has been reported that somatic loss of function of the tumor suppressor *tuberous sclerosis 2* (*TSC-2)* leads to the development of benign and malignant lesions in the myometrium, kidney and other tissues sharing common features such as a low rate of renewal and defects in the mitochondrial respiratory chain associated with oncogenesis [41,42]. This gene is annotated in all the functional categories prevalent in the IC subset. Intriguingly, it is predicted that *TSC-2* mRNA interacts strongly with Nuclear Protein 5A (NOP56). The interaction propensity is 175 corresponding to an AUC of 99.5%. This protein is an essential component of the splicing machinery [43] that is differentially expressed in leiomyoma and downregulated in response to hypoxia [44]. It is possible that hypoxia-dependent repression of NOP56 expression [45-47] is a protective mechanism against fast growth and potential tumor progression. Indeed, it has been reported that *NOP56* and *TSC-2* are not differentially expressed in renal carcinomas and oncocytomas [48,49] (ArrayExpress: E-GEOD-12090; ArrayExpress: E-GEOD-19982), indicating loss of regulation during malignant progression.

Based on these observations, we propose that downregulation of RBPs promoting the translation of dysfunctional tumor suppressors can prevent indiscriminate cellular growth and that loss of control can destine a cell to malignancy (additional examples are reported in Additional file 1).

### Anti-expression and interaction propensity are features of repressing processes

**Figure 5:**
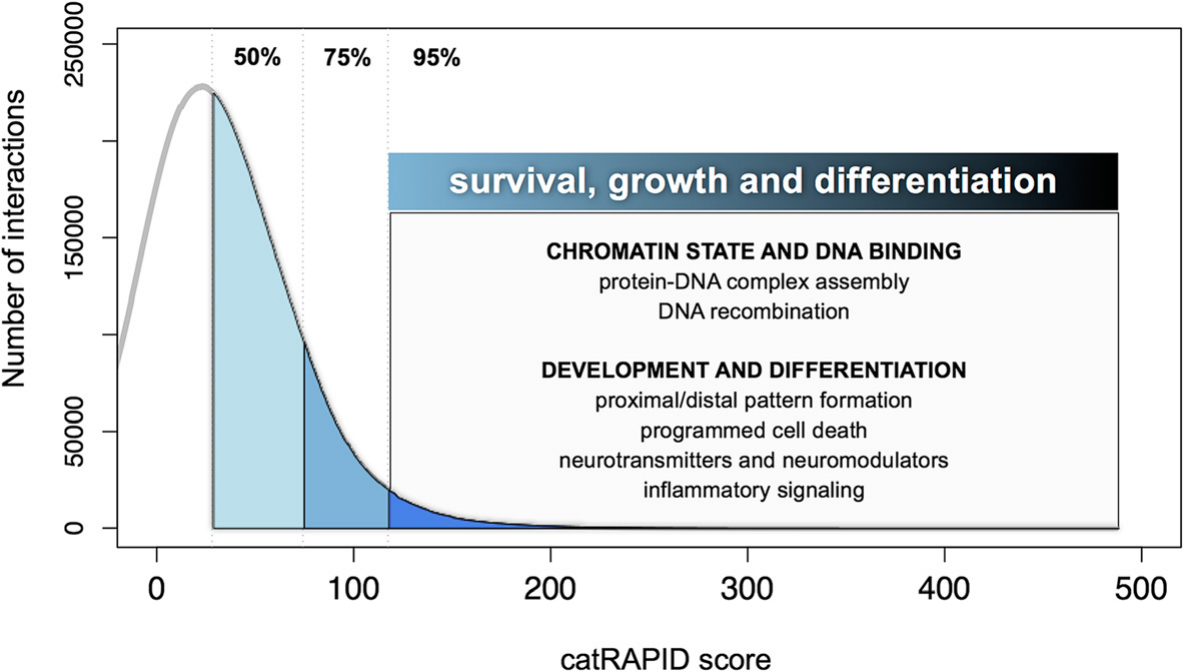
**GO enrichment for interacting mRNA–RBP pairs anti-correlated in expression (IA subset).** Using the catRAPID score distribution, we evaluated mRNA GO enrichment associated with different areas under the curve (see Materials and methods). A color gradient (cyan to blue) shows the AUC values (number of interactions: 20,702,804 for AUC > 50%, 10,351,402 for AUC > 75%, 2,070,280 for AUC > 95%). We found that cell differentiation processes are more prevalent in interacting mRNA–RBP pairs (AUC > 95%) that are anti-correlated in expression. AUC, area under the curve; GO, gene ontology; IA, interacting and anti-expressed; RBP, RNA-binding protein.

For AUC > 95%, the IA subset has more terms associated with cell differentiation processes (for example, proximal/distal pattern formation) as well as inflammation (for example, positive regulation of isotype switching), which are known to be tightly linked [50-52]. In fact, a number of differentiation cytokines (IL18, IL23 and EBI3/IL27) and stimulators of cytokine production (CD28 and CD80CCR2/CD192) are in the subset. Moreover, a large fraction of entries is also linked to protein–DNA complex assembly and regulation of transcription initiation from RNA polymerase II promoter (Figure 5 and Additional file 3: Table S1). It has been shown that 94% of genes in IA enriched functional categories (124 out of 132) are listed in the annotated gene index of the National Institutes of Health’s Cancer Genome Anatomy Project [40]. Remarkably, terms clearly associated with cell differentiation and inflammation (especially regulation of embryonic development and B cell activation involved in immune response) are more prevalent in the IA subset when immunochemistry data are used.

IA genes share the common functional property of regulating survival, growth and differentiation processes. As RBPs play a crucial role in repressing gene expression [53,54], IA associations could be involved in the regulation of proliferative events. Indeed, adult tissues are constantly maintained at the steady state [13] but a dramatic reawakening of growth, survival and differentiation genes occur in either physiological conditions (for example, wound healing [50]) or pathological progression to cancer [55].

In the IA set, we found YTHDC1 (YT521-B), which is a ubiquitously expressed member of the novel RNA-binding YTH-domain family [56]. YTHDC1 represses gene expression by either sequestering splicing factors or directly binding to transcripts [57-59] (Additional file 2: Figure S5A). Among the transcripts that we predict to be potentially targeted by YTHDC1, we found several proto-oncogenes or tumor-associated genes such as *RET, PRMT2, RARG and HOXA9* (*RET*: interaction propensity = 166; *PRMT2*: interaction propensity = 209; *RARG*: interaction propensity = 194; *HOXA9*: interaction propensity = 165; all corresponding to an AUC of 99.5%). In particular, alternatively spliced variants of *PRMT2* were related to survival and the invasiveness of breast cancer cells [60,61], while high expression of *RARG* and *HOXA9* has been observed in human hepatocellular carcinomas and acute leukemia [62,63]. We hypothesize that perturbation of the regulation by YTHDC1 of potentially oncogenic genes such as *RET, PRMT2, RARG and HOXA9* could be involved in the pathogenesis of related tumors. In fact, experimental studies support the implications for YTHDC1 in cancer progression with regard to angiogenesis, growth factor signaling, immortalization, genetic instability, tissue invasion and apoptosis [59,64,65].

Similarly, the translational silencer TIA-1, also reported to induce mRNA decay [66-68], is predicted to interact with the ubiquitously expressed *NAP1L1* transcript (interaction propensity = 113 corresponding to an AUC of 95%), consistent with iCLIP data for HeLa cells (ArrayExpress: E-MTAB-432) [69] (Additional file 4: Table S2). Deregulation of *NAP1L1* expression has been documented for several tumors such as small intestine carcinoid neoplasia [70], neuroendocrine tumors [71], ovarian cancer [72] and hepatoblastomas [73]. We hypothesize that TIA-1 plays a fundamental role in the post-transcriptional regulation of *NAP1L1* and that alteration of this regulatory process contributes to *NAP1L1-*associated tumor development.

We note that repression of aberrant interactions can be achieved by gene silencing, which prevents the potential stabilizing action of RBPs on specific transcripts (Additional file 2: Figure S5B). For instance, the *Nodal* gene is normally silenced in adult tissues and its expression is associated with tumor progression [74]. Since Nodal is a member of the Transforming Growth Factor β (TGFB) superfamily and controls mesoderm formation and axial patterning during embryonic development [74], it is possible that *Nodal* interactions with specific RBPs lead to pathogenesis in adult tissues. Our predictions indicate that the transcript *Nodal* interacts with a number of anti-expressed RBPs (ADD1, API5, ARCN1, CANX, CAPRIN1, CCT6A, DKFZP434I0812, GSPT1, HSP90AB1, PKM, PUF60, XRCC5, YTHDC1 and YWHAZ). Since the exact mechanism regulating *Nodal* is at present unknown, we generated a list of protein partners that could be exploited for future experimental studies (Additional file 5: Table S3).

## Conclusions

Comparative expression studies provide important insights into biological processes and can lead to the discovery of unknown regulation patterns. While evolutionary constraints on tissue-specific gene expression patterns have been extensively investigated [7-9,75,76], the constitutive regulation of RBP-mediated interactions is still poorly understood [11,12]. It has been previously observed that cellular localization and gene expression levels impose stringent conditions on the physicochemical properties of both protein and RNA sequences [77,78], but large-scale computational analyses of constitutive RBP-mediated regulatory networks have never been attempted before. Our study shows for the first time that the integration of *in silico* predictions [19] with *ex vivo* expression profile data [6,34] can be used to discover distinct features of RBP biological functions.

We observed an enrichment of unique and functionally related GO terms for RBP–mRNA pairs associated with high interaction propensities and specific expression patterns. In our analysis, co-expression of interacting mRNA–RBP pairs (IC set) is linked to regulation of proliferation and cell cycle control, while anti-expression (IA set) is a characteristic feature of survival, growth and differentiation-specific processes. We do not exclude that RBP–mRNA associations displaying poor interaction propensities (NIC and NIA sets) might have important evolutionary implications as spatiotemporal separation and limited chemical reactivity could be ways to avoid aberrant associations [55].

We found that RNA-binding proteins are enriched in structurally disordered regions and that unfolded polypeptide fragments promote association with RNA molecules at low interaction propensities. As disordered proteins are highly reactive [37], it is reasonable to assume that interaction with RNA needs to be tightly regulated to avoid cellular damage [39]. In this regard, our results expand at the nucleic acid level what has been previously observed for the general promiscuity of natively unfolded proteins [38,79].

In conclusion, we hope that our study of protein–RNA interaction and expression will be useful in the design of new experiments and for further characterizing ribonucleoprotein associations. A list of proposed interactions and a server for new inquiries are available at the catRAPID express webpage [27].

## Materials and methods

### Prediction for LIN28B and HuR interactions

We performed a number of tests to assess the quality of our calculations (see section on RNA-binding protein–mRNA interaction propensity) using PAR-CLIP data [31,33]. In this analysis, we used all the RNA interactions present in our dataset (positive set: 285 sequences for LIN28B and 579 for HuR) and, due to the unavailability of non-bound RNAs, the full list of human transcripts (negative set: 105,000 sequences).

For the s*ignificance of interaction predictions*, we performed Fisher’s exact test comparing the top 1% of predicted interactions with the remaining protein–RNA associations (HuR: *P* = 10^-8^; LIN28B: *P* = 10^-3^). Fisher’s exact test was computed using equal amounts (that is, 1% of the total interactions) of randomly extracted negative subsets (HuR: *P* = 10^-7^; LIN28B: *P* = 0.0002; Additional file 2: Figure S3).

*For the significance of score distributions*, we used Student’s *t*-test to compare the score distribution of positives and negatives (HuR: *P* = 10^-16^; LIN28B: *P* = 10^-4^). We also performed Student’s *t*-test using random extractions of negative subsets, each containing the same number of RNAs as positives (LIN28B: *P* = 0.03; HuR: *P* < 10^-8^; Student’s *t*-test).

Other statistical tests (receiver operating characteristics and precision/recall curves) are discussed in Additional file 1. The expression data for HuR and LIN28B were taken from the original manuscripts [31,33] and processed as indicated by the authors. The datasets were downloaded from GEO [29] (GSE29943) and ArrayExpress [80] (E-GEOD-44615 and E-GEOD-44613).

### mRNA dataset: Human BodyMap

The Human BodyMap (HBM) 2.0 contains expression data generated using the Hiseq 2000 system and it has expression profiles for a number of human tissues [22]. The HBM RNA sequencing (RNA-seq) data was downloaded from ArrayExpress [81] under accession number E-MTAB-513. The final mRNA dataset contained 35,818 transcripts (11,584 genes) with expression levels for 14 human tissues (see section on RNA-binding protein–mRNA expression). We considered all human cDNAs from EnsEMBL release 68. Transcripts incompatible with the catRAPID size restrictions (that is, 50 to 1,200 nucleotides) or not expressed in at least one tissue were filtered out. In the analysis, we evaluated different CD-HIT [82] sequence similarity cutoff thresholds (see section on Gene ontology analysis).

### RNA-binding protein dataset: Human Protein Atlas

We considered all the RBPs reported in two studies on RBPs binding to mRNAs [1,2]. The initial dataset consisted of 3,500 RBPs (832 genes). Proteins incompatible with catRAPID’s size restrictions (that is, 50 to 750 amino acids) and above a CD-HIT [82] sequence similarity cutoff of 75% were filtered out. Similarly, proteins not present in the Human Protein Atlas (HPA) database (version 11.0) [34] and not expressed in at least one tissue were discarded. The final RBP (HPA) dataset contained 612 proteins (491 genes) with expression levels for 14 human tissues (see section on RNA-binding protein–mRNA expression). All protein sequences were retrieved from EnsEMBL release 68.

### RNA-binding protein dataset: Human BodyMap

As for RBPs in the HPA, filters on sequence size and redundancy were applied. Proteins not present in the Human BodyMap database (version 2.0) [6] were discarded. The final RBP (HBM) dataset contained 1,156 proteins (543 genes) with expression levels for 14 human tissues (see section on RNA-binding protein–mRNA expression). All protein sequences were retrieved from EnsEMBL release 68.

### RNA-binding protein–mRNA expression

We analyzed 14 human tissues for which both immunohistochemistry [34] and transcript abundances [6] were available. At present, the Human Protein Atlas is the largest collection of protein abundance data available [34]. Transcripts in the *mRNA dataset* and proteins in the *RBP dataset* were represented by vectors containing the normalized relative abundance of the following tissues: adrenal gland, brain, breast, colon, heart, kidney, liver, lung, lymph, muscle, lymph node, ovary, prostate and thyroid. For the immunohistochemistry data*,* the read-outs ‘no’, ‘low’, ‘intermediate’ or ‘high’ expression were transformed into numbers (0, 1, 2, 3) and subject to *Z*-normalization per tissue. As for the transcript data, the vectors were Z-normalized using the average and standard deviation per tissue. For each RBP–mRNA combination we computed the pairwise Pearson’s correlation coefficient of the vectors. As shown in Additional file 2: Figures S1 and S2, we observed the same trends using immunohistochemistry [34] and RNA-seq data [6] to estimate protein abundances in human tissues.

### RNA-binding protein–mRNA interaction propensity

We used catRAPID [19,20] to compute the interaction propensity of each protein in the RBP dataset with each transcript in the mRNA dataset. catRAPID predicts protein–RNA associations by estimating the interaction propensity between amino acids and nucleotides using secondary structure information, hydrogen bonding and Van der Waals forces [19,20]. The approach was previously applied to predict associations between different types of proteins and RNA molecules [21,23]. Although each protein binds to distinct types of RNA structures [83], we observe that the contribution of hairpin loops accounts for 57% of the overall interaction propensity [19]. The catRAPID web server is publicly accessible from our webpage [84].

### Protein–RNA interaction and expression

For a given protein, interacting (*n*_*int*_) and non-interacting (*n*_*no-int*_) protein–RNA pairs were compared at different AUCs (areas under the curve) of the interaction propensity distribution. The enrichment in positively correlated expression (Figure 2C) is calculated as:

(1) $$\begin{array}{l} \mathrm{enrichment}\;\left(\mathrm{co}‒\mathrm{expressed}\;\mathrm{interactions}\right)\\ \;=\frac{n_{\mathrm{int}}\;\left(r>r_{\mathrm{th}}\right)-n_{\mathrm{no}‒\mathrm{int}}\;\left(r>r_{\mathrm{th}}\right)}{n_{\mathrm{no}‒\mathrm{int}}\;\left(r>r_{\mathrm{th}}\right)} \end{array}$$

In Equation (1), the correlation coefficient *r* follows the distribution of protein–RNA expression and the parameter *r*_*th*_ > 0 corresponds to an AUC spanning the range 50% to 99.5% (Figure 2B).

Similarly, for negatively correlated expressions (Figure 2D):

(2) $$\begin{array}{l} \mathrm{enrichment}\;\left(\mathrm{anti}‒\mathrm{expressed}\;\mathrm{interactions}\right)\\ \;=\frac{n_{\mathrm{int}}\left(r<l_{\mathrm{th}}\right)-n_{\mathrm{no}‒\mathrm{int}}\left(r<l_{\mathrm{th}}\right)}{n_{\mathrm{no}‒\mathrm{int}}\left(r<l_{\mathrm{th}}\right)} \end{array}$$

In Equation (2), the parameter *l*_*th*_ < 0 corresponds to an AUC spanning the range 50% to 99.5% (Figure 2B).

### Gene ontology analysis

For each area under the curve (AUC) of the catRAPID score distribution (50% < *AUC* < 99.5%), we created four subsets according to the correlation in tissue expression: (1) IC subset: positively correlating and interacting genes (expression correlation ≥ +0.7 and positive interaction propensities); (2) IA subset: negatively correlating and interacting genes (expression correlation ≤ −0.7 and positive interaction propensities); (3) NIC subset: positively correlating and non-interacting genes (expression correlation ≥ + 0.7 and negative interaction propensities); (4) NIA subset: negatively correlating and non-interacting genes (expression correlation ≤ −0.7 and negative interaction propensities). The expression correlation of |0.7| corresponds to AUC = 95% of the statistical distribution, for which we found the highest enrichments (Figure 2C,D). We systematically applied conditional tests for GO term over-representation in each subset using the GOStats package (version 2.28.0) available from Bioconductor [85]. To assess the over-representation of a GO term in one particular subset at a certain AUC, we considered five criteria (Additional file 3: Table S1; Additional file 6: Table S4; Additional file 2: Figure S6):

1. The GO term must be reported for more than two genes.

2. The *P* value of the GO term must be significant (*P* < 0.05) in the subset of interest and non-significant (*P* > 0.1) in the others.

3. The enrichment must be conserved with respect to: (a) the entire human transcriptome (that is, including RNAs longer than 1,200 nucleotides and independently of expression data), (b) the complete set of analyzed genes (that is, including RNAs shorter than 1,200 nucleotides and with available expression) and (c) all genes under the same AUC (that is, considering both interacting and non-interacting pairs at the two tails of the distribution).

4. The *P* value of the GO term must be non-significant (*P* > 0.1) in: (a) the complete set of analyzed genes compared to the human transcriptome (significance would indicate enrichment irrespective of the subset assignment) and (b) the list of transcripts compatible with catRAPID length requirements compared to the human transcriptome (significance would indicate length bias in the statistics; see section on length bias statistics).

5. The enrichment must be conserved after sequence redundancy reduction to the 80% identity threshold.

### Length bias statistics

Due to the conformational space of nucleotide chains, prediction of RNA secondary structures is difficult when RNA sequences are >1,200 nucleotides and simulations cannot be completed on standard processors (2.5 GHz; 4 to 8 GB memory). To see whether GO enrichment is biased by the catRAPID length restriction, we used a hypergeometric test (see section on the RNA-binding protein–mRNA interaction propensity). If a GO term is enriched in the length-restricted set, it is excluded *a priori* from the analysis because genes annotated in that GO term would be only selected for the length range. Thus, we imposed that GO terms must be non-significant (*P* > 0.1) in the length-restricted set of genes (see section on gene ontology analysis). This condition ensures that there is no bias due to length restrictions for any GO term enriched in a particular subset (Additional file 3: Table S1).

### Analysis of RNA-binding protein sequence disorder

The content of disordered regions in the RBP sequences was computed using IUPred [36]. For each protein, we extracted structurally disordered regions (IUPred score higher than 0.4) and calculated their interactions against the reference transcriptome. We compared the interaction propensities of each disordered region with that of the full-length protein and assessed if there was an increase or decrease of the interaction propensity score (Figure 3A). The contribution of the disordered region was evaluated using a Mann–Whitney U test, where a significant increase (*P* < 0.05; H_0_ < H_1_) in the interaction propensity score is associated with a positive contribution. From low to high interaction propensities, the contribution of disorder decreases progressively with respect to that of the full-length proteins (Figure 3A). The role of disorder is more pronounced in proteins lacking canonical RNA-binding domains, indicating that unstructured regions have a direct involvement in contacting RNA (Figure 3B).

### Web server

catRAPID express [27] is a publicly available implementation of catRAPID [19,20], which is used to study the relation between protein–RNA interaction propensity and expression in *Homo sapiens*. The tool has two components: (1) catRAPID predictions of protein–RNA interaction and (2) the computation of correlation using protein and RNA expression profiles [6,34]. A description of how catRAPID makes predictions can be found in the *Documentation*, *Tutorial* and *Frequently Asked Questions* (*FAQs*) on the webpage. Expression profiles of the RBP dataset and mRNA dataset are assigned respectively to input proteins and RNA using a homology-based criterion (ten top-ranked proteins with a BLAST [86]*e* ≤ 0.01 and ≥75% whole sequence similarity; ten top-ranked transcripts with a BLAST *e* ≤ 0.01 and ≥95% whole sequence similarity). Sequence similarity is evaluated using the Needleman–Wunsch algorithm [87].

## Abbreviations

AUC: area under the curve; GEO: Gene Expression Omnibus; GO: gene ontology; HBM: Human BodyMap; HPA: Human Protein Atlas; HuR: human antigen R; IA: interacting and anti-expressed; IC: interacting and co-expressed; LFC: log-fold change; LIN28B: lin-28 homolog B; NIA: non-interacting and anti-expressed; NIC: non-interacting and co-expressed; NOP56: Nuclear Protein 5A; PAR-CLIP: photoactivatable-ribonucleoside-enhanced cross-linking and immunoprecipitation; RBP: RNA-binding protein; RNA-seq: RNA sequencing; TSC-2: tuberous sclerosis 2.

## Competing interests

The authors declare that there are no competing financial interests.

## Authors’ contributions

GGT conceived this study. FA and GGT designed the *in silico* experiments. DC, FA and CML performed the computational analysis. DC, DM, TBO, FA and GGT analyzed the data. DM, DC and CML searched the literature on RNA networks. DC, DM and GGT wrote the manuscript. All authors read and approved the final version of the manuscript.

## Supplementary Material

Additional file 1 Additional materials and methods. Click here for file

Additional file 2: Figure S1 With respect to non-interacting protein–RNA pairs, non-correlated protein–RNA expression does not show enrichment using **(A)** RNA and **(B)** protein expression. Areas under the curve (AUCs) were used to select the same number of interacting/non-interacting and positively/negatively expressed protein–RNA pairs for the analysis. **Figure S2.** Protein–RNA interaction and expression (immunohistochemistry expression data). **(A)** With respect to non-interacting protein–RNA pairs, predicted associations had enriched positively correlated expression. **(B)** Compared to non-interacting protein–RNA pairs, predicted associations had enriched negatively correlated expression. **Figure S3.***P* value distribution for HuR and LIN28B predictions. We compared *P* values (Fisher’s exact test) for the catRAPID predictions for HuR and LIN28B RNA interactions (red arrow) using balanced bootstrap resampling (random extractions of negative subsets with the same amount as the positive subset). The predicted interactions differ significantly from random associations. **Figure S4.** Receiver operating characteristic (ROC) and precision/recall (PR) curves for HuR and LIN28B predictions. We evaluated changes in the ROC and PR curves for the catRAPID predictions for the **(A)** HuR and **(B)** LIN28B RNA interactome for random samples using several ratios of positive and negative associations (pos/neg ratios). **Figure S5.** Examples of protein–RNA anti-expression scenarios. **(A)** We propose that YTHDC1 represses the expression of tumor-associated genes by destabilizing mRNAs. **(B)***Nodal* expression in adult tissues is associated with tumor progression, which might be due to transcript stabilization. **Figure S6.** Nested representation of gene sets used in GO enrichment analysis. **Figure S7.** Changes in transcript and gene counts after sequence redundancy reduction. The mRNA database comprises 35,818 transcripts (11,584 genes). After redundancy filtering, the mRNA database is reduced to 33,936 transcripts (11,483 genes) at 95% sequence identity threshold; 32,700 transcripts (11,406 genes) at 90%; 31,287 transcripts (11360 genes) at 85% and 29,673 transcripts (11,317 genes) at 80%. Click here for file

Additional file 3: Table S1 mRNA GO-term enrichment analysis of interacting mRNA–RBP pairs (*P* values). Every GO term has been tested for over-representation for each subset (IC, IA, NIC and NIA) with respect to the human transcriptome, the complete set of analyzed genes (analyzed mRNA set) and the analyzed genes with the same AUC (relative AUC subset). Statistical control for the biased over-representation of GO terms in the complete set of analyzed genes and in a catRAPID length-restricted set of genes was used for the human transcriptome (see Materials and methods). Significant *P* values for the IC and IA subsets are shown in red. GOBP, gene ontology biological process; GOCC, gene ontology cellular component; GOMF, gene ontology molecular function; IA, interacting and anti-correlated in expression; IC, interacting and correlated in expression; NIC, not interacting and correlated in expression; Seq%id, sequence identity threshold used for redundancy reduction. Click here for file

Additional file 4: Table S2 *NAP1L1* read counts from TIA-1 iCLIP data. The count of reads mapping into the *NAP1L1* gene and the relative cumulative distribution functions (cdf) are reported from the iCLIP experiment for controls and replicates of the TIA-1 protein [20]. The read count cdf was estimated after removal of genes with zero counts. Click here for file

Additional file 5: Table S3 *Nodal* anti-expressed interacting (IA) RBPs. Click here for file

Additional file 6: Table S4 mRNA GO-term enrichment analysis of interacting mRNA–RBP pairs (IC and IA gene counts are reported). Every GO term has been tested for over-representation in the IC and IA subsets with respect to the human transcriptome, the complete set of analyzed genes (analyzed mRNAs set) and the analyzed genes under the same AUC (relative AUC subset).GOBP, gene ontology biological process; GOCC, gene ontology cellular component; GOMF, gene ontology molecular function; IA, interacting and anti-correlated in expression; IC, interacting and correlated in expression; Seq%id, sequence identity threshold used for redundancy reduction. Click here for file

## References

[B1] Castello A Fischer B Eichelbaum K Horos R Beckmann BM Strein C Davey NE Humphreys DT Preiss T Steinmetz LM Krijgsveld J Hentze MW Insights into RNA biology from an atlas of mammalian mRNA-binding proteins Cell 2012 149 1393 1406 10.1016/j.cell.2012.04.031 22658674

[B2] Baltz AG Munschauer M Schwanhäusser B Vasile A Murakawa Y Schueler M Youngs N Penfold-Brown D Drew K Milek M Wyler E Bonneau R Selbach M Dieterich C Landthaler M The mRNA-bound proteome and its global occupancy profile on protein-coding transcripts Mol Cell 2012 46 674 690 10.1016/j.molcel.2012.05.021 22681889

[B3] Siomi H Dreyfuss G RNA-binding proteins as regulators of gene expression Curr Opin Genet Dev 1997 7 345 353 10.1016/S0959-437X(97)80148-7 9229110

[B4] Cook KB Kazan H Zuberi K Morris Q Hughes TR RBPDB: a database of RNA-binding specificities Nucleic Acids Res 2011 39 D301 D308 10.1093/nar/gkq1069 21036867 PMC3013675

[B5] Dassi E Malossini A Re A Mazza T Tebaldi T Caputi L Quattrone A AURA: Atlas of UTR Regulatory Activity Bioinforma Oxf Engl 2012 28 142 144 10.1093/bioinformatics/btr608 22057158

[B6] Harrow J Frankish A Gonzalez JM Tapanari E Diekhans M Kokocinski F Aken BL Barrell D Zadissa A Searle S Barnes I Bignell A Boychenko V Hunt T Kay M Mukherjee G Rajan J Despacio-Reyes G Saunders G Steward C Harte R Lin M Howald C Tanzer A Derrien T Chrast J Walters N Balasubramanian S Pei B Tress M GENCODE: the reference human genome annotation for The ENCODE Project Genome Res 2012 22 1760 1774 10.1101/gr.135350.111 22955987 PMC3431492

[B7] Merkin J Russell C Chen P Burge CB Evolutionary dynamics of gene and isoform regulation in mammalian tissues Science 2012 338 1593 1599 10.1126/science.1228186 23258891 PMC3568499

[B8] Brawand D Soumillon M Necsulea A Julien P Csárdi G Harrigan P Weier M Liechti A Aximu-Petri A Kircher M Albert FW Zeller U Khaitovich P Grützner F Bergmann S Nielsen R Pääbo S Kaessmann H The evolution of gene expression levels in mammalian organs Nature 2011 478 343 348 10.1038/nature10532 22012392

[B9] Chan ET Quon GT Chua G Babak T Trochesset M Zirngibl RA Aubin J Ratcliffe MJH Wilde A Brudno M Morris QD Hughes TR Conservation of core gene expression in vertebrate tissues J Biol 2009 8 33 10.1186/jbiol130 19371447 PMC2689434

[B10] Wittkopp PJ Haerum BK Clark AG Evolutionary changes in cis and trans gene regulation Nature 2004 430 85 88 10.1038/nature02698 15229602

[B11] Masuda K Kuwano Y Nishida K Rokutan K General RBP expression in human tissues as a function of age Ageing Res Rev 2012 11 423 431 10.1016/j.arr.2012.01.005 22326651

[B12] Hogan DJ Riordan DP Gerber AP Herschlag D Brown PO Diverse RNA-binding proteins interact with functionally related sets of RNAs. Suggesting an extensive regulatory system PLoS Biol 2008 6 e255 10.1371/journal.pbio.0060255 18959479 PMC2573929

[B13] Vogel C Marcotte EM Insights into the regulation of protein abundance from proteomic and transcriptomic analyses Nat Rev Genet 2012 13 227 232 22411467 10.1038/nrg3185 PMC3654667

[B14] Maier T Güell M Serrano L Correlation of mRNA and protein in complex biological samples FEBS Lett 2009 583 3966 3973 10.1016/j.febslet.2009.10.036 19850042

[B15] Tartaglia GG Pechmann S Dobson CM Vendruscolo M Life on the edge: a link between gene expression levels and aggregation rates of human proteins Trends Biochem Sci 2007 32 204 206 10.1016/j.tibs.2007.03.005 17419062

[B16] Greenbaum D Colangelo C Williams K Gerstein M Comparing protein abundance and mRNA expression levels on a genomic scale Genome Biol 2003 4 117 10.1186/gb-2003-4-9-117 12952525 PMC193646

[B17] Cox B Kislinger T Emili A Integrating gene and protein expression data: pattern analysis and profile mining Methods San Diego Calif 2005 35 303 314 10.1016/j.ymeth.2004.08.021 15722226

[B18] Greenbaum D Jansen R Gerstein M Analysis of mRNA expression and protein abundance data: an approach for the comparison of the enrichment of features in the cellular population of proteins and transcripts Bioinforma Oxf Engl 2002 18 585 596 10.1093/bioinformatics/18.4.585 12016056

[B19] Bellucci M Agostini F Masin M Tartaglia GG Predicting protein associations with long noncoding RNAs Nat Methods 2011 8 444 445 10.1038/nmeth.1611 21623348

[B20] Cirillo D Agostini F Tartaglia GG Predictions of protein–RNA interactions Wiley Interdiscip Rev Comput Mol Sci 2013 3 161 175 10.1002/wcms.1119

[B21] Agostini F Cirillo D Bolognesi B Tartaglia GG X-inactivation: quantitative predictions of protein interactions in the Xist network Nucleic Acids Res 2013 41 e31 10.1093/nar/gks968 23093590 PMC3592426

[B22] Iglesias-Platas I Martin-Trujillo A Cirillo D Court F Guillaumet-Adkins A Camprubi C Bourc’his D Hata K Feil R Tartaglia G Arnaud P Monk D Characterization of novel paternal ncRNAs at the Plagl1 locus, including Hymai, predicted to interact with regulators of active chromatin PLoS One 2012 7 e38907 10.1371/journal.pone.0038907 22723905 PMC3378578

[B23] Cirillo D Agostini F Klus P Marchese D Rodriguez S Bolognesi B Tartaglia GG Neurodegenerative diseases: quantitative predictions of protein–RNA interactions RNA 2013 19 129 140 10.1261/rna.034777.112 23264567 PMC3543085

[B24] Johnson R Noble W Tartaglia GG Buckley NJ Neurodegeneration as an RNA disorder Prog Neurobiol 2012 99 293 315 10.1016/j.pneurobio.2012.09.006 23063563 PMC7116994

[B25] Zanzoni A Marchese D Agostini F Bolognesi B Cirillo D Botta-Orfila M Livi CM Rodriguez-Mulero S Tartaglia GG Principles of self-organization in biological pathways: a hypothesis on the autogenous association of alpha-synuclein Nucleic Acids Res 2013 41 9987 9998 10.1093/nar/gkt794 24003031 PMC3905859

[B26] Agostini F Zanzoni A Klus P Marchese D Cirillo D Tartaglia GG catRAPID omics: a web server for large-scale prediction of protein–RNA interactions Bioinformatics 2013 29 2928 2930 10.1093/bioinformatics/btt495 23975767 PMC3810848

[B27] catRAPID express [http://service.tartaglialab.com/page/catrapid_express_group]

[B28] Ashburner M Ball CA Blake JA Botstein D Butler H Cherry JM Davis AP Dolinski K Dwight SS Eppig JT Harris MA Hill DP Issel-Tarver L Kasarskis A Lewis S Matese JC Richardson JE Ringwald M Rubin GM Sherlock G Gene ontology: tool for the unification of biology. The gene ontology consortium Nat Genet 2000 25 25 29 10.1038/75556 10802651 PMC3037419

[B29] Barrett T Wilhite SE Ledoux P Evangelista C Kim IF Tomashevsky M Marshall KA Phillippy KH Sherman PM Holko M Yefanov A Lee H Zhang N Robertson CL Serova N Davis S Soboleva A NCBI GEO: archive for functional genomics data sets – update Nucleic Acids Res 2012 41 D991 D995 23193258 10.1093/nar/gks1193 PMC3531084

[B30] Parkinson H Kapushesky M Kolesnikov N Rustici G Shojatalab M Abeygunawardena N Berube H Dylag M Emam I Farne A Holloway E Lukk M Malone J Mani R Pilicheva E Rayner TF Rezwan F Sharma A Williams E Bradley XZ Adamusiak T Brandizi M Burdett T Coulson R Krestyaninova M Kurnosov P Maguire E Neogi SG Rocca-Serra P Sansone S-A ArrayExpress update – from an archive of functional genomics experiments to the atlas of gene expression Nucleic Acids Res 2009 37 D868 D872 10.1093/nar/gkn889 19015125 PMC2686529

[B31] Lebedeva S Jens M Theil K Schwanhäusser B Selbach M Landthaler M Rajewsky N Transcriptome-wide analysis of regulatory interactions of the RNA-binding protein HuR Mol Cell 2011 43 340 352 10.1016/j.molcel.2011.06.008 21723171

[B32] Graf R Munschauer M Mastrobuoni G Mayr F Heinemann U Kempa S Rajewsky N Landthaler M Identification of LIN28B-bound mRNAs reveals features of target recognition and regulation RNA Biol 2013 10 1146 1159 10.4161/rna.25194 23770886 PMC3849162

[B33] Hafner M Max KEA Bandaru P Morozov P Gerstberger S Brown M Molina H Tuschl T Identification of mRNAs bound and regulated by human LIN28 proteins and molecular requirements for RNA recognition RNA 2013 19 613 626 10.1261/rna.036491.112 23481595 PMC3677277

[B34] Uhlen M Oksvold P Fagerberg L Lundberg E Jonasson K Forsberg M Zwahlen M Kampf C Wester K Hober S Wernerus H Björling L Ponten F Towards a knowledge-based human protein atlas Nat Biotechnol 2010 28 1248 1250 10.1038/nbt1210-1248 21139605

[B35] Lu P Vogel C Wang R Yao X Marcotte EM Absolute protein expression profiling estimates the relative contributions of transcriptional and translational regulation Nat Biotechnol 2007 25 117 124 10.1038/nbt1270 17187058

[B36] Dosztányi Z Csizmok V Tompa P Simon I IUPred: web server for the prediction of intrinsically unstructured regions of proteins based on estimated energy content Bioinforma Oxf Engl 2005 21 3433 3434 10.1093/bioinformatics/bti541 15955779

[B37] Gsponer J Babu MM Cellular strategies for regulating functional and nonfunctional protein aggregation Cell Rep 2012 2 1425 1437 10.1016/j.celrep.2012.09.036 23168257 PMC3607227

[B38] Babu MM van der Lee R de Groot NS Gsponer J Intrinsically disordered proteins: regulation and disease Curr Opin Struct Biol 2011 21 432 440 10.1016/j.sbi.2011.03.011 21514144

[B39] Vavouri T Semple JI Garcia-Verdugo R Lehner B Intrinsic protein disorder and interaction promiscuity are widely associated with dosage sensitivity Cell 2009 138 198 208 10.1016/j.cell.2009.04.029 19596244

[B40] Strausberg RL Buetow KH Emmert-Buck MR Klausner RD The cancer genome anatomy project: building an annotated gene index Trends Genet TIG 2000 16 103 106 10.1016/S0168-9525(99)01937-X 10689348

[B41] Cai S Everitt JI Kugo H Cook J Kleymenova E Walker CL Polycystic kidney disease as a result of loss of the tuberous sclerosis 2 tumor suppressor gene during development Am J Pathol 2003 162 457 468 10.1016/S0002-9440(10)63840-0 12547704 PMC1851170

[B42] Simonnet H Demont J Pfeiffer K Guenaneche L Bouvier R Brandt U Schagger H Godinot C Mitochondrial complex I is deficient in renal oncocytomas Carcinogenesis 2003 24 1461 1466 10.1093/carcin/bgg109 12844484

[B43] Wahl MC Will CL Lührmann R The spliceosome: design principles of a dynamic RNP machine Cell 2009 136 701 718 10.1016/j.cell.2009.02.009 19239890

[B44] Crabtree JS Jelinsky SA Harris HA Choe SE Cotreau MM Kimberland ML Wilson E Saraf KA Liu W McCampbell AS Dave B Broaddus RR Brown EL Kao W Skotnicki JS Abou-Gharbia M Winneker RC Walker CL Comparison of human and rat uterine leiomyomata: identification of a dysregulated mammalian target of rapamycin pathway Cancer Res 2009 69 6171 6178 10.1158/0008-5472.CAN-08-4471 19622772

[B45] Francia G Man S Teicher B Grasso L Kerbel RS Gene expression analysis of tumor spheroids reveals a role for suppressed DNA mismatch repair in multicellular resistance to alkylating agents Mol Cell Biol 2004 24 6837 6849 10.1128/MCB.24.15.6837-6849.2004 15254249 PMC444854

[B46] Manalo DJ Rowan A Lavoie T Natarajan L Kelly BD Ye SQ Garcia JGN Semenza GL Transcriptional regulation of vascular endothelial cell responses to hypoxia by HIF-1 Blood 2005 105 659 669 10.1182/blood-2004-07-2958 15374877

[B47] Beyer S Kristensen MM Jensen KS Johansen JV Staller P The histone demethylases JMJD1A and JMJD2B are transcriptional targets of hypoxia-inducible factor HIF J Biol Chem 2008 283 36542 36552 10.1074/jbc.M804578200 18984585 PMC2662309

[B48] Rohan S Tu JJ Kao J Mukherjee P Campagne F Zhou XK Hyjek E Alonso MA Chen Y-T Gene expression profiling separates chromophobe renal cell carcinoma from oncocytoma and identifies vesicular transport and cell junction proteins as differentially expressed genes Clin Cancer Res Off J Am Assoc Cancer Res 2006 12 6937 6945 10.1158/1078-0432.CCR-06-1268 17145811

[B49] Tan M-H Wong CF Tan HL Yang XJ Ditlev J Matsuda D Khoo SK Sugimura J Fujioka T Furge KA Kort E Giraud S Ferlicot S Vielh P Amsellem-Ouazana D Debré B Flam T Thiounn N Zerbib M Benoît G Droupy S Molinié V Vieillefond A Tan PH Richard S Teh BT Genomic expression and single-nucleotide polymorphism profiling discriminates chromophobe renal cell carcinoma and oncocytoma BMC Cancer 2010 10 196 10.1186/1471-2407-10-196 20462447 PMC2883967

[B50] Martin P Parkhurst SM Parallels between tissue repair and embryo morphogenesis Dev Camb Engl 2004 131 3021 3034 10.1242/dev.01253 15197160

[B51] Lu H Ouyang W Huang C Inflammation, a key event in cancer development Mol Cancer Res MCR 2006 4 221 233 10.1158/1541-7786.MCR-05-0261 16603636

[B52] Rider CC Mulloy B Bone morphogenetic protein and growth differentiation factor cytokine families and their protein antagonists Biochem J 2010 429 1 12 10.1042/BJ20100305 20545624

[B53] Standart N Jackson RJ Regulation of translation by specific protein/mRNA interactions Biochimie 1994 76 867 879 10.1016/0300-9084(94)90189-9 7880904

[B54] De Moor CH Richter JD Translational control in vertebrate development Int Rev Cytol 2001 203 567 608 11131527 10.1016/s0074-7696(01)03017-0

[B55] Quenneville S Turelli P Bojkowska K Raclot C Offner S Kapopoulou A Trono D The KRAB-ZFP/KAP1 system contributes to the early embryonic establishment of site-specific DNA methylation patterns maintained during development Cell Rep 2012 2 766 773 10.1016/j.celrep.2012.08.043 23041315 PMC3677399

[B56] Zhang Z Theler D Kaminska KH Hiller M de la Grange P Pudimat R Rafalska I Heinrich B Bujnicki JM Allain FH-T Stamm S The YTH domain is a novel RNA binding domain J Biol Chem 2010 285 14701 14710 10.1074/jbc.M110.104711 20167602 PMC2863249

[B57] Harigaya Y Tanaka H Yamanaka S Tanaka K Watanabe Y Tsutsumi C Chikashige Y Hiraoka Y Yamashita A Yamamoto M Selective elimination of messenger RNA prevents an incidence of untimely meiosis Nature 2006 442 45 50 10.1038/nature04881 16823445

[B58] Rafalska I Zhang Z Benderska N Wolff H Hartmann AM Brack-Werner R Stamm S The intranuclear localization and function of YT521-B is regulated by tyrosine phosphorylation Hum Mol Genet 2004 13 1535 1549 10.1093/hmg/ddh167 15175272

[B59] Zhang B Zur Hausen A Orlowska-Volk M Jäger M Bettendorf H Stamm S Hirschfeld M Yiqin O Tong X Gitsch G Stickeler E Alternative splicing-related factor YT521: an independent prognostic factor in endometrial cancer Int J Gynecol Cancer Off J Int Gynecol Cancer Soc 2010 20 492 499 10.1111/IGC.0b013e3181d66ffe 20686370

[B60] Baldwin RM Morettin A Paris G Goulet I Côté J Alternatively spliced protein arginine methyltransferase 1 isoform PRMT1v2 promotes the survival and invasiveness of breast cancer cells Cell Cycle 2012 11 4597 4612 10.4161/cc.22871 23187807 PMC3562305

[B61] Zhong J Cao R-X Zu X-Y Hong T Yang J Liu L Xiao X-H Ding W-J Zhao Q Liu J-H Wen G-B Identification and characterization of novel spliced variants of PRMT2 in breast carcinoma FEBS J 2012 279 316 335 10.1111/j.1742-4658.2011.08426.x 22093364

[B62] Yan T-D Wu H Zhang H-P Lu N Ye P Yu F-H Zhou H Li W-G Cao X Lin Y-Y He J-Y Gao W-W Zhao Y Xie L Chen J-B Zhang X-K Zeng J-Z Oncogenic potential of retinoic acid receptor-gamma in hepatocellular carcinoma Cancer Res 2010 70 2285 2295 10.1158/0008-5472.CAN-09-2968 20197465

[B63] Li D-P Li Z-Y Sang W Cheng H Pan X-Y Xu K-L HOXA9 gene expression in acute myeloid leukemia Cell Biochem Biophys 2013 67 935 938 10.1007/s12013-013-9586-8 23575938

[B64] Hirschfeld M Zhang B Jaeger M Stamm S Erbes T Mayer S Tong X Stickeler E Hypoxia-dependent mRNA expression pattern of splicing factor YT521 and its impact on oncological important target gene expression Mol Carcinog 2013 in press 10.1002/mc.22045 23765422

[B65] Harris AL Hypoxia – a key regulatory factor in tumour growth Nat Rev Cancer 2002 2 38 47 10.1038/nrc704 11902584

[B66] Piecyk M Wax S Beck AR Kedersha N Gupta M Maritim B Chen S Gueydan C Kruys V Streuli M Anderson P TIA-1 is a translational silencer that selectively regulates the expression of TNF-alpha EMBO J 2000 19 4154 4163 10.1093/emboj/19.15.4154 10921895 PMC306595

[B67] Dixon DA Balch GC Kedersha N Anderson P Zimmerman GA Beauchamp RD Prescott SM Regulation of cyclooxygenase-2 expression by the translational silencer TIA-1 J Exp Med 2003 198 475 481 10.1084/jem.20030616 12885872 PMC2194089

[B68] Yamasaki S Stoecklin G Kedersha N Simarro M Anderson P T-cell intracellular antigen-1 (TIA-1)-induced translational silencing promotes the decay of selected mRNAs J Biol Chem 2007 282 30070 30077 10.1074/jbc.M706273200 17711853

[B69] Wang Z Kayikci M Briese M Zarnack K Luscombe NM Rot G Zupan B Curk T Ule J iCLIP predicts the dual splicing effects of TIA–RNA interactions PLoS Biol 2010 8 e1000530 10.1371/journal.pbio.1000530 21048981 PMC2964331

[B70] Kidd M Modlin IM Mane SM Camp RL Eick G Latich I The role of genetic markers – NAP1L1, MAGE-D2, and MTA1 – in defining small-intestinal carcinoid neoplasia Ann Surg Oncol 2006 13 253 262 10.1245/ASO.2006.12.011 16424981

[B71] Drozdov I Kidd M Nadler B Camp RL Mane SM Hauso O Gustafsson BI Modlin IM Predicting neuroendocrine tumor (carcinoid) neoplasia using gene expression profiling and supervised machine learning Cancer 2009 115 1638 1650 10.1002/cncr.24180 19197975 PMC2743551

[B72] Guidi F Puglia M Gabbiani C Landini I Gamberi T Fregona D Cinellu MA Nobili S Mini E Bini L Modesti PA Modesti A Messori L 2D-DIGE analysis of ovarian cancer cell responses to cytotoxic gold compounds Mol Biosyst 2012 8 985 993 10.1039/c1mb05386h 22134777

[B73] Nagata T Takahashi Y Ishii Y Asai S Nishida Y Murata A Koshinaga T Fukuzawa M Hamazaki M Asami K Ito E Ikeda H Takamatsu H Koike K Kikuta A Kuroiwa M Watanabe A Kosaka Y Fujita H Miyake M Mugishima H Transcriptional profiling in hepatoblastomas using high-density oligonucleotide DNA array Cancer Genet Cytogenet 2003 145 152 160 10.1016/S0165-4608(03)00065-7 12935928

[B74] Lawrence MG Margaryan NV Loessner D Collins A Kerr KM Turner M Seftor EA Stephens CR Lai J Postovit L-M Clements JA Hendrix MJC APC BioResource Reactivation of embryonic nodal signaling is associated with tumor progression and promotes the growth of prostate cancer cells Prostate 2011 71 1198 1209 10.1002/pros.21335 21656830 PMC3234312

[B75] Ravasi T Suzuki H Cannistraci CV Katayama S Bajic VB Tan K Akalin A Schmeier S Kanamori-Katayama M Bertin N Carninci P Daub CO Forrest ARR Gough J Grimmond S Han J-H Hashimoto T Hide W Hofmann O Kamburov A Kaur M Kawaji H Kubosaki A Lassmann T van Nimwegen E MacPherson CR Ogawa C Radovanovic A Schwartz A Teasdale RD An atlas of combinatorial transcriptional regulation in mouse and man Cell 2010 140 744 752 10.1016/j.cell.2010.01.044 20211142 PMC2836267

[B76] Wu L Candille SI Choi Y Xie D Jiang L Li-Pook-Than J Tang H Snyder M Variation and genetic control of protein abundance in humans Nature 2013 499 79 82 10.1038/nature12223 23676674 PMC3789121

[B77] Tartaglia GG Vendruscolo M Correlation between mRNA expression levels and protein aggregation propensities in subcellular localisations Mol Biosyst 2009 5 1873 1876 10.1039/b913099n 19763336

[B78] Tartaglia GG Pechmann S Dobson CM Vendruscolo M A relationship between mRNA expression levels and protein solubility in *E. coli* J Mol Biol 2009 388 381 389 10.1016/j.jmb.2009.03.002 19281824

[B79] Olzscha H Schermann SM Woerner AC Pinkert S Hecht MH Tartaglia GG Vendruscolo M Hayer-Hartl M Hartl FU Vabulas RM Amyloid-like aggregates sequester numerous metastable proteins with essential cellular functions Cell 2011 144 67 78 10.1016/j.cell.2010.11.050 21215370

[B80] ArrayExpress [http://www.ebi.ac.uk/arrayexpress]

[B81] ArrayExpress [http://www.ebi.ac.uk/arrayexpress/experiments/E-MTAB-513]

[B82] Li W Godzik A Cd-hit: a fast program for clustering and comparing large sets of protein or nucleotide sequences Bioinformatics 2006 22 1658 1659 10.1093/bioinformatics/btl158 16731699

[B83] Li X Kazan H Lipshitz HD Morris QD Finding the target sites of RNA-binding proteins Wiley Interdiscip Rev RNA 2014 5 111 130 10.1002/wrna.1201 24217996 PMC4253089

[B84] Tartaglia’s group web servers [http://service.tartaglialab.com]

[B85] Falcon S Gentleman R Using GOstats to test gene lists for GO term association Bioinforma Oxf Engl 2007 23 257 258 10.1093/bioinformatics/btl567 17098774

[B86] Altschul SF Gish W Miller W Myers EW Lipman DJ Basic local alignment search tool J Mol Biol 1990 215 403 410 2231712 10.1016/S0022-2836(05)80360-2

[B87] Rice P Longden I Bleasby A EMBOSS: the European molecular biology open software suite Trends Genet TIG 2000 16 276 277 10.1016/S0168-9525(00)02024-2 10827456

