# Supplementary material for: Constitutive patterns of gene expression regulated by RNA-binding proteins
Source: Genome Biol. 2014 Jan 2;15(1):R13. doi: 10.1186/gb-2014-15-1-r13 (PMC4054784; doi:10.1186/gb-2014-15-1-r13)
Supplement: Additional file 1 — Additional materials and methods. [file gb-2014-15-1-r13-S1.pdf]

Additional Materials and Methods

Constitutive patterns of gene expression regulated by RNA-binding proteins

Davide Cirillo[1,2,$], Domenica Marchese[1,2,$], Federico Agostini[1,2], Carmen Maria Livi[1,2], Teresa Botta-Orfila[1,2], Gian Gaetano Tartaglia[1,2]

[1]Gene Function and Evolution, Centre for Genomic Regulation (CRG), Dr. Aiguader 88, 08003 Barcelona, Spain, [2]Universitat Pompeu Fabra (UPF), 08003 Barcelona, Spain.

Corresponding author: Gian Gaetano Tartaglia.

Telephone: +34 933160116.

Email: gian.tartaglia@crg.eu.

[$] These authors contributed equally to the work.

Overview of the results

The mechanisms through which RBPs integrate different *stimuli* and respond to metabolic needs can be very diverse:

1. *positive correlation in expression profiles*: RBPs can promote the expression of a transcript by stabilizing the mRNA and enhancing its recruitment to the translational machinery (e.g., elongation and export to the cytoplasm).

2. *negative correlation in expression profiles*: RBPs can negatively regulate gene expression by blocking RNA polyadenylation and transport or inducing its sequestration and degradation.

3. *positive interaction propensities:* RBPs can exert a direct effect on RNA metabolism by physically interacting with the transcript through sequence or structural recognition motifs.

4. *negative interaction propensities*: RBPs do not play any role or can indirect regulate the expression of other effectors associated with RNA processing.

Coexpression and interaction propensity are features of cell cycle control

We note that genes coding for inhibitors of CDKs show particularly high interaction propensities. In agreement with experimental evidence [1], we found that the quaking homolog RNA-binding protein QKI, belonging to the STAR (signal transduction and activation of RNA) family, is co-expressed with *p27* as well as *MBP* and binds strongly to them (*p27*: interaction propensity = 65.57 corresponding to an AUC of 63%; discriminative power = 98%. *MBP*: interaction propensity = 70.64 corresponding to an AUC of 69%; discriminative power = 98%). Interestingly, protein p27 is a cell cycle inhibitor and is related to activation of the *MBP* promoter [2].

Anti-expression and interaction propensity are features of differentiation

In our analysis, the anti-expression of RBP and mRNA reflects the precise spatio-temporal dynamics of interactions associated with growth, survival and differentiation. Although the current level of knowledge provides only a limited basis for validation, we found a number of highly interacting and anti-correlating RBP-mRNA associations that can be linked to repression of differentiation processes in adult tissues. Some illustrative examples are reported below.

Non-phosphorylated YTHDC1 localizes in nuclear *foci* termed YT bodies (markers for differentiated cells)[3] where YTHDC1 interacts with the spliceosome assembly mediator Sam68 (KHDRBS1)[4]. A significant impact of YTHDC1 on tumorigenesis has been recently demonstrated: a clear association of low YTHDC1 expression with poor clinical outcome in patients diagnosed with type I endometrial cancer was identified, demonstrating a potential tumor suppressor activity [5]. Alterations of YTHDC1 expression might have eminent impact on splicing decision of cancer-associated genes [6]. Such regulatory mechanisms can be proposed for *PBR* and *LINGO1* genes, negative regulators of oligodendrocytes proliferation and differentiation, that were found up-regulated in a number of neuropathologies, including gliomas and neurodegenerative disorders in the case of *PBR* [7], and in substantia nigra of Parkinson's disease patients in the case of *LINGO1* [8].

Non-correlated protein-RNA interaction and expression

For non-correlated expressions (Figure S1A), the following formula is used:

$$enrichment\ (no - expression) = \frac{n_{int}(l_{th}<r<r_{th}) - n_{no-int}(l_{th}<r<r_{th})}{n_{no-int}(l_{th}<r<r_{th})} \quad (1)$$

In Eq. (1), l_{th} and r_{th} values correspond to positive and negative correlation coefficients of AUCs taken from the protein-RNA expression distribution.

The same trends were observed using either immunohistochemistry [9] or RNA sequencing data (Supplementary Figure 1B).

Prediction of LIN28B and HUR interactions

Extra analysis for the examples reported in this paper are reported below:

- When comparing 500 top- and 500 bottom-scored interactions (highest and lowest interaction propensities), the area under the receiver operating characteristics (ROC) curve is 0.69 (LIN28B: 0.71; HUR: 0.67).
- If the same number of positives and negatives are extracted randomly from the database and the procedure is repeated 10 times, the area under the ROC curve is 0.66 (LIN28B: 0.67; HUR: 0.66; Supplementary Figure 4A).
- If the number of positives is unbalanced with respect to the number of negatives (i.e. for 1 positive there are 10 negatives / 1 negative for 10 positives) the area under the ROC curve is 0.64 (LIN28B: 0.64 / 0.63; HUR: 0.64 / 0.64; Supplementary Figure 4A). In this test, positives and negatives are extracted randomly and the procedure is repeated 10 times. If for 1 positive 100 negatives are randomly extracted, the area under the ROC curve is 0.63 (LIN28B: 0.63; HUR: 0.62; Supplementary Figure 4A).

In addition, we performed Precision/Recall (PR) analysis (Supplementary Figure 4B):

- When the same number of positives and negatives are randomly extracted from the databases and the procedure is repeated 10 times, the area under the PR curve is 0.61 (LIN28B: 0.62; HUR: 0.60).
- If there are 10 positives for each negative, the area under the PR curve is 0.91 (LIN28B: 0.94; HUR: 0.93).
- If there are 10 negatives for each positive, the area under the PR curve is 0.34 (LIN28B: 0.13; HUR: 0.16).

- If there are 100 negatives for each positive, the area under the PR curve is 0.02 (LIN28B: 0.02; HUR: 0.02).

The PR analysis indicates that performances depend on the size of the sampling space, as reported in previous studies [10]. Indeed, the asymptotic limit of the PR curve is dominated by the number of false positives and tends to zero as the number of negatives increases. In case of rare events (small clusters to be identified in large amount of data) PR curves show limited variability, as shown in previous analyses [11]. It should be noted that the area under the ROC size only weakly depends on the dataset size, in agreement with what has been previously shown [10].

We compared our performances with those achievable by considering the occurrence of recognition motifs reported in literature. In these calculations, we used position weighted matrices [12] and FIMO [13] with p-value threshold of 0.05:

- When the same number of positives and negatives are randomly extracted from the databases and the procedure is repeated 10 times, the area under the ROC curve is 0.53 (LIN28B: 0.52; HUR: 0.54), while the area under the PR curve is 0.53 (LIN28B: 0.51; HUR: 0.55).
- If there are 10 positives for each negative, the area under the ROC curve is 0.58 (LIN28B: 0.56; HUR: 0.60), while the area under the PR curve is 0.90 (LIN28B: 0.91; HUR: 0.90).
- If there are negatives for each positive, the area under the ROC curve is 0.53 (LIN28B: 0.52; HUR: 0.54), while the area under the PR curve is 0.11 (LIN28B: 0.10; HUR: 0.12).
- If there are 100 negatives for each positive, the area under the ROC curve is 0.51 (LIN28B: 0.52; HUR: 0.54), while the area under the PR curve is 0.01 (LIN28B: 0.01; HUR: 0.01).

Hence, our results show that catRAPID outperforms the motif search. It is important to mention that HUR and LIN28B targets are reported in the original papers [16, 17], but non-interacting RNAs are difficult to obtain. In fact, we do not exclude that there are transcripts in the control sets that are able to bind to HUR or LIN28B (even if their expression levels are low in the cell line of the experimental study).

References

1. Biedermann B, Hotz H-R, Ciosk R: **The Quaking family of RNA-binding proteins: coordinators of the cell cycle and differentiation**. *Cell Cycle* 2010, **9**:1929–1933.

2. Wei Q, Miskimins WK, Miskimins R: **Sox10 acts as a tissue-specific transcription factor enhancing activation of the myelin basic protein gene promoter by p27Kip1 and Sp1**. *J Neurosci Res* 2004, **78**:796–802.

3. Nayler O, Hartmann AM, Stamm S: **The ER repeat protein YT521-B localizes to a novel subnuclear compartment**. *J Cell Biol* 2000, **150**:949–962.

4. Hartmann AM, Nayler O, Schwaiger FW, Obermeier A, Stamm S: **The interaction and colocalization of Sam68 with the splicing-associated factor YT521-B in nuclear dots is regulated by the Src family kinase p59(fyn)**. *Mol Biol Cell* 1999, **10**:3909–3926.

5. Zhang B, zur Hausen A, Orlowska-Volk M, Jäger M, Bettendorf H, Stamm S, Hirschfeld M, Yiqin O, Tong X, Gitsch G, Stickeler E: **Alternative splicing-related factor YT521: an independent prognostic factor in endometrial cancer**. *Int J Gynecol Cancer* 2010, **20**:492–499.

6. Hirschfeld M, Zhang B, Jaeger M, Stamm S, Erbes T, Mayer S, Tong X, Stickeler E: **Hypoxia-dependent mRNA expression pattern of splicing factor YT521 and its impact on oncological important target gene expression**. *Mol Carcinog* 2013.

7. Papadopoulos V, Lecanu L, Brown RC, Han Z, Yao Z-X: **Peripheral-type benzodiazepine receptor in neurosteroid biosynthesis, neuropathology and neurological disorders**. *Neuroscience* 2006, **138**:749–756.

8. Inoue H, Lin L, Lee X, Shao Z, Mendes S, Snodgrass-Belt P, Sweigard H, Engber T, Pepinsky B, Yang L, Beal MF, Mi S, Isacson O: **Inhibition of the leucine-rich repeat protein LINGO-1 enhances survival, structure, and function of dopaminergic neurons in Parkinson's disease models**. *Proc Natl Acad Sci USA* 2007, **104**:14430–14435.

9. Uhlen M, Oksvold P, Fagerberg L, Lundberg E, Jonasson K, Forsberg M, Zwahlen M, Kampf C, Wester K, Hober S, Wernerus H, Björling L, Ponten F: **Towards a knowledge-based Human Protein Atlas**. *Nat Biotechnol* 2010, **28**:1248–1250.

10. Fawcett T: **An introduction to ROC analysis**. *Pattern Recognition Letters* 2006, **27**:861–874.

11. Krzyzanowski PM, Andrade-Navarro MA: **Identification of novel stem cell markers using gap analysis of gene expression data**. *Genome Biology* 2007, **8**:R193.

12. Ray D, Kazan H, Cook KB, Weirauch MT, Najafabadi HS, Li X, Gueroussov S, Albu M, Zheng H, Yang A, Na H, Irimia M, Matzat LH, Dale RK, Smith SA, Yarosh CA, Kelly SM, Nabet B, Mecenas D, Li W, Laishram RS, Qiao M, Lipshitz HD, Piano F, Corbett AH, Carstens RP, Frey BJ, Anderson RA, Lynch KW, Penalva LOF, et al.: **A compendium of RNA-binding motifs for decoding gene regulation**. *Nature* 2013, **499**:172–177.

13. Grant CE, Bailey TL, Noble WS: **FIMO: scanning for occurrences of a given motif**. *Bioinformatics* 2011, **27**:1017–1018.

14. Wilbert ML, Huelga SC, Kapeli K, Stark TJ, Liang TY, Chen SX, Yan BY, Nathanson JL, Hutt KR, Lovci MT, Kazan H, Vu AQ, Massirer KB, Morris Q, Hoon S, Yeo GW: **LIN28 binds messenger RNAs at GGAGA motifs and regulates splicing factor abundance**. *Mol Cell* 2012, **48**:195–206.

15. Li X, Kazan H, Lipshitz HD, Morris QD: **Finding the target sites of RNA-binding proteins**. *Wiley Interdiscip Rev RNA* 2013.

16. Lebedeva S, Jens M, Theil K, Schwanhäusser B, Selbach M, Landthaler M, Rajewsky N: **Transcriptome-wide Analysis of Regulatory Interactions of the RNA-Binding Protein HuR**. *Molecular Cell* 2011, **43**:340–352.

17. Hafner M, Max KEA, Bandaru P, Morozov P, Gerstberger S, Brown M, Molina H, Tuschl T: **Identification of mRNAs bound and regulated by human LIN28 proteins and molecular requirements for RNA recognition**. *RNA* 2013, **19**:613–626.

18. Harigaya Y, Tanaka H, Yamanaka S, Tanaka K, Watanabe Y, Tsutsumi C, Chikashige Y, Hiraoka Y, Yamashita A, Yamamoto M: **Selective elimination of messenger RNA prevents an incidence of untimely meiosis**. *Nature* 2006, **442**:45–50.

19. Lawrence MG, Margaryan NV, Loessner D, Collins A, Kerr KM, Turner M, Seftor EA, Stephens CR, Lai J, APC BioResource, Postovit L-M, Clements JA, Hendrix MJC: **Reactivation of embryonic nodal signaling is associated with tumor progression and promotes the growth of prostate cancer cells**. *Prostate* 2011, **71**:1198–1209.

20. Wang Z, Kayikci M, Briese M, Zarnack K, Luscombe NM, Rot G, Zupan B, Curk T, Ule J: **iCLIP predicts the dual splicing effects of TIA-RNA interactions**. *PLoS Biol* 2010, **8**:e1000530.
