# Supplementary material for: Constitutive patterns of gene expression regulated by RNA-binding proteins
Source: Genome Biol. 2014 Jan 2;15(1):R13. doi: 10.1186/gb-2014-15-1-r13 (PMC4054784; doi:10.1186/gb-2014-15-1-r13)
Supplement: Additional file 2: Figure S1 — With respect to non-interacting protein–RNA pairs, non-correlated protein–RNA expression does not show enrichment using (A) RNA and (B) protein expression. Areas under the curve (AUCs) were used to select the same number of interacting/non-interacting and positively/negatively expressed protein–RNA pairs for the analysis. Figure S2. Protein–RNA interaction and expression (immunohistochemistry expression data). (A) With respect to non-interacting protein–RNA pairs, predicted associations had enriched positively correlated expression. (B) Compared to non-interacting protein–RNA pairs, predicted associations had enriched negatively correlated expression. Figure S3.P value distribution for HuR and LIN28B predictions. We compared P values (Fisher’s exact test) for the catRAPID predictions for HuR and LIN28B RNA interactions (red arrow) using balanced bootstrap resampling (random extractions of negative subsets with the same amount as the positive subset). The predicted interactions differ significantly from random associations. Figure S4. Receiver operating characteristic (ROC) and precision/recall (PR) curves for HuR and LIN28B predictions. We evaluated changes in the ROC and PR curves for the catRAPID predictions for the (A) HuR and (B) LIN28B RNA interactome for random samples using several ratios of positive and negative associations (pos/neg ratios). Figure S5. Examples of protein–RNA anti-expression scenarios. (A) We propose that YTHDC1 represses the expression of tumor-associated genes by destabilizing mRNAs. (B)Nodal expression in adult tissues is associated with tumor progression, which might be due to transcript stabilization. Figure S6. Nested representation of gene sets used in GO enrichment analysis. Figure S7. Changes in transcript and gene counts after sequence redundancy reduction. The mRNA database comprises 35,818 transcripts (11,584 genes). After redundancy filtering, the mRNA database is reduced to 33,936 transcripts (11,483 genes) at 95% sequence ide [file gb-2014-15-1-r13-S2.pdf]

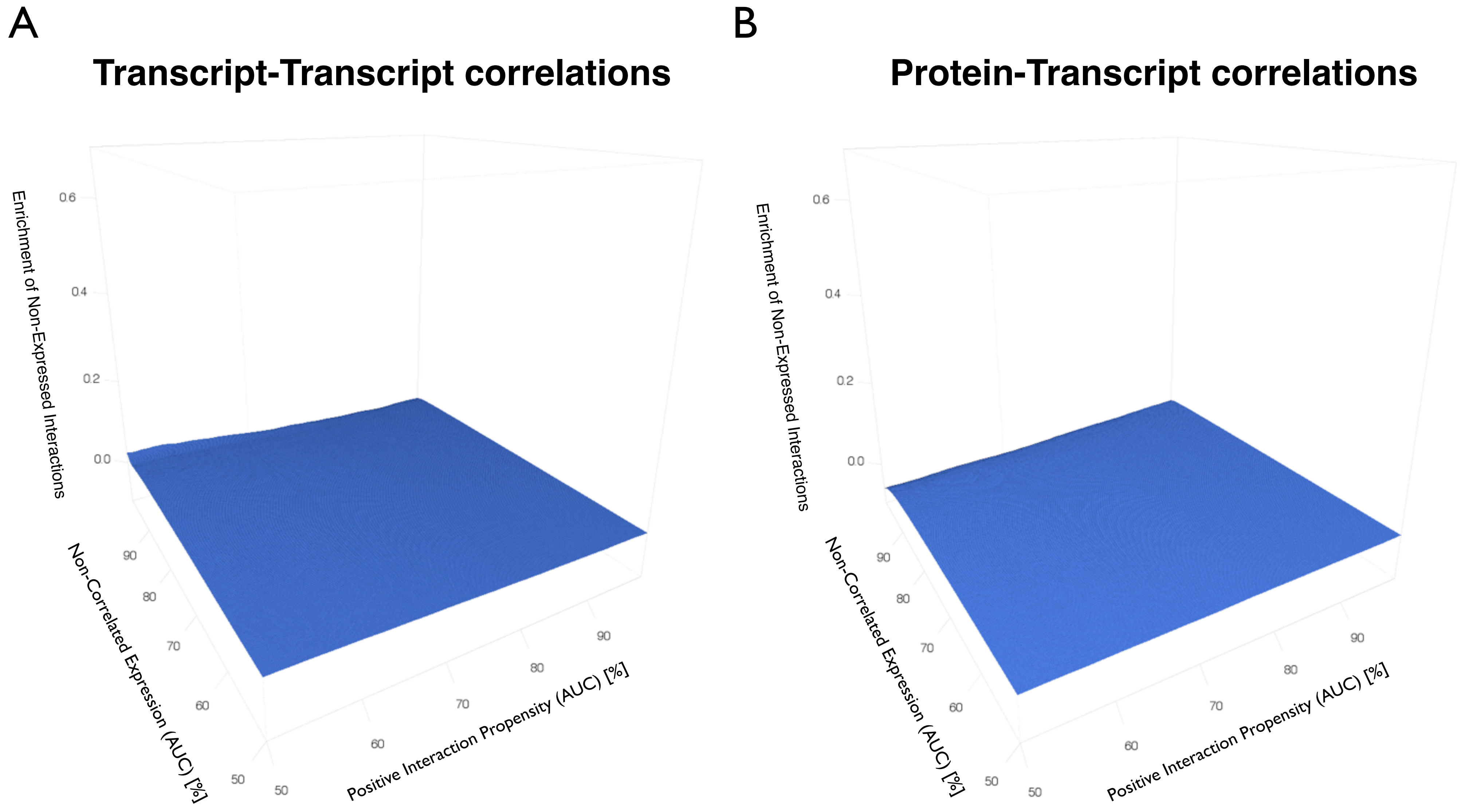
A
Transcript-Transcript correlations
Enrichment of Non-Expressed Interactions
0.6
0.4
0.2
0.0
Non-Correlated Expression (AUC) [%]
90
80
70
60
50
Positive Interaction Propensity (AUC) [%]
50
60
70
80
90
B
Protein-Transcript correlations
Enrichment of Non-Expressed Interactions
0.6
0.4
0.2
0.0
Non-Correlated Expression (AUC) [%]
90
80
70
60
50
Positive Interaction Propensity (AUC) [%]
50
60
70
80
90

Figure S1

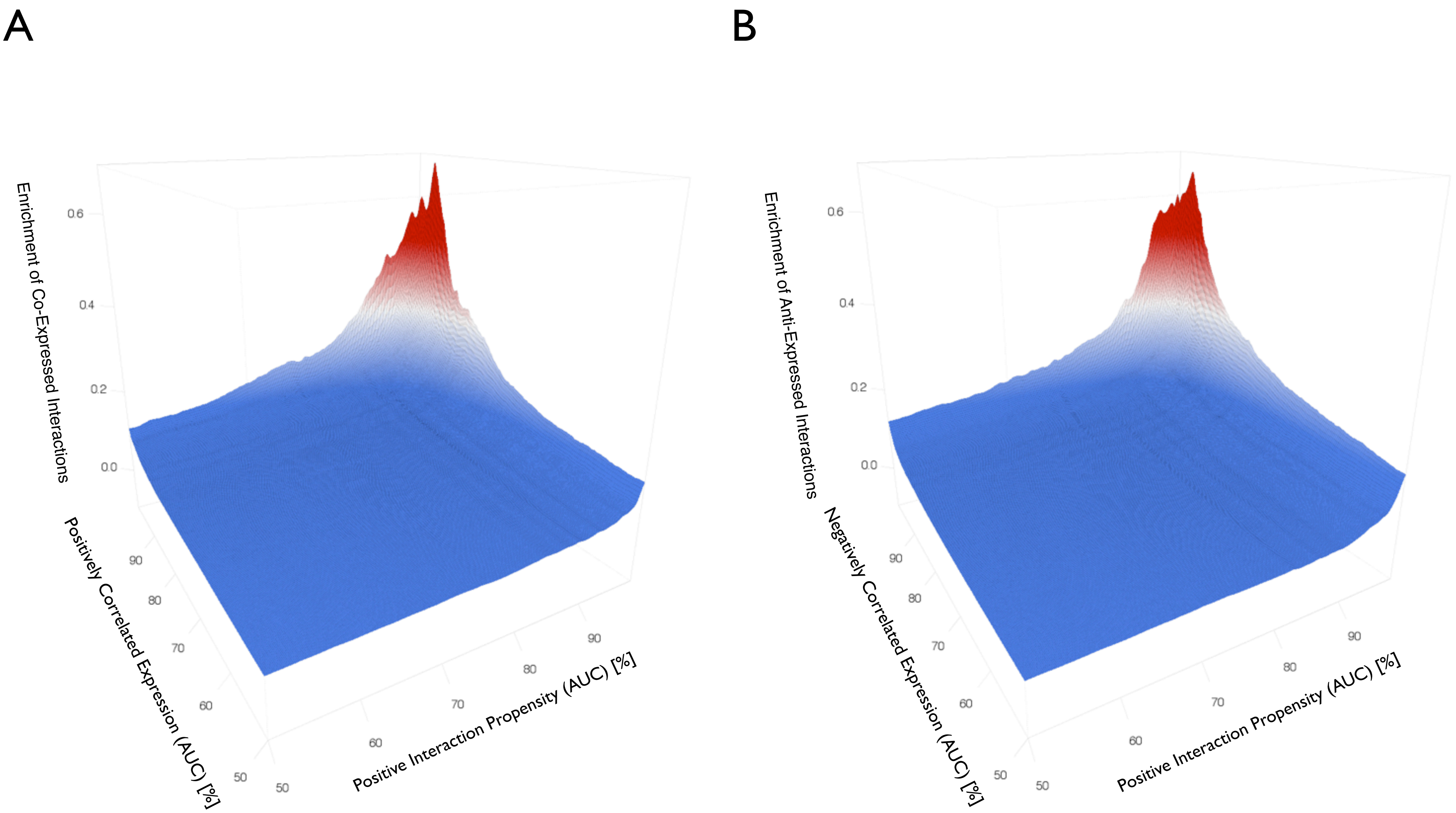

A
Enrichment of Co-Expressed Interactions
0.6
0.4
0.2
0.0
Positively Correlated Expression (AUC) [%]
90
80
70
60
50
50
60
70
80
90
Positive Interaction Propensity (AUC) [%]
B
Enrichment of Anti-Expressed Interactions
0.6
0.4
0.2
0.0
Negatively Correlated Expression (AUC) [%]
90
80
70
60
50
50
60
70
80
90
Positive Interaction Propensity (AUC) [%]

Figure S2

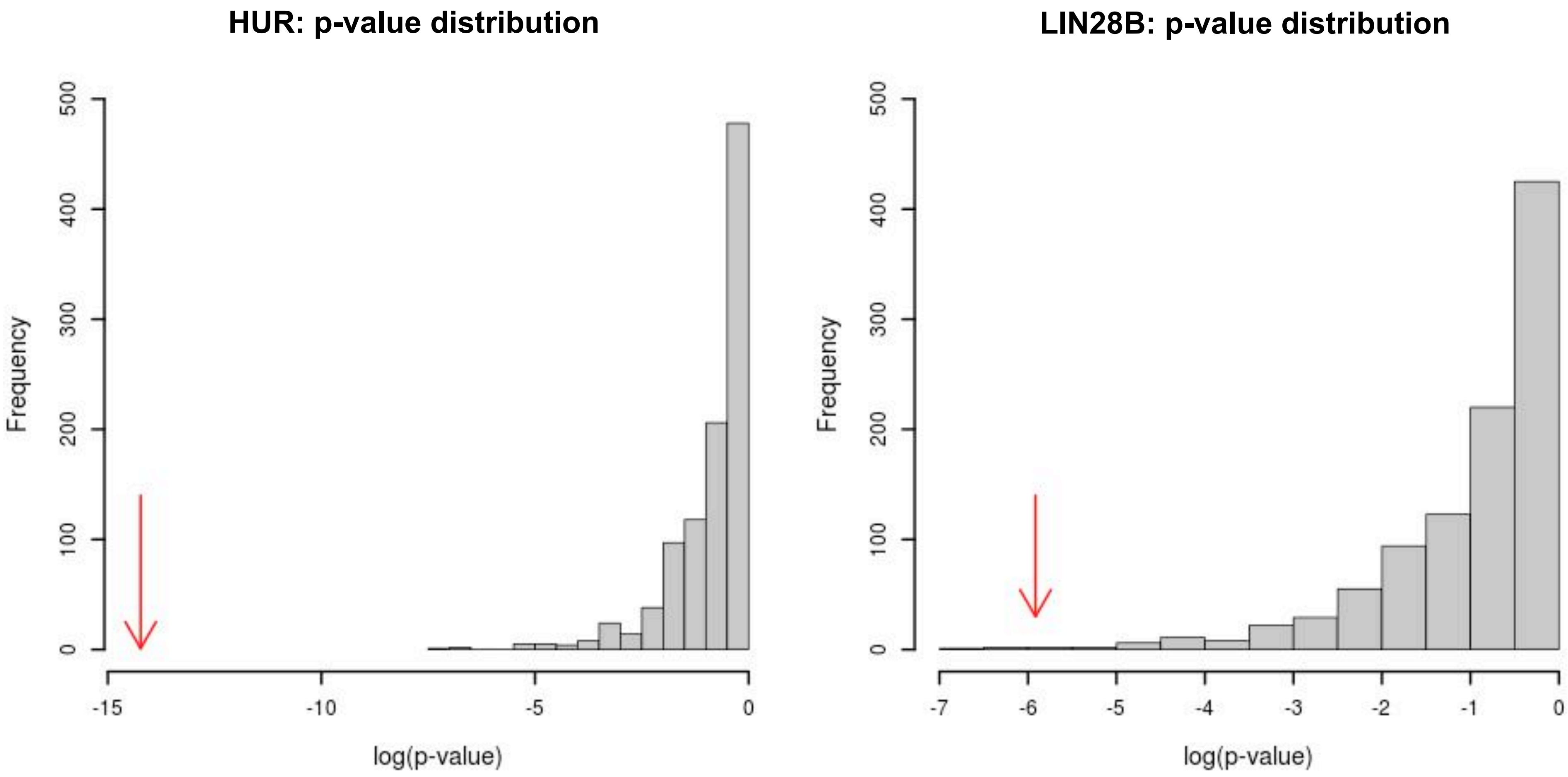
HUR: p-value distribution
Frequency
500
400
300
200
100
0
-15
-10
-5
0
log(p-value)
LIN28B: p-value distribution
Frequency
500
400
300
200
100
0
-7
-6
-5
-4
-3
-2
-1
0
log(p-value)

Figure S3

A

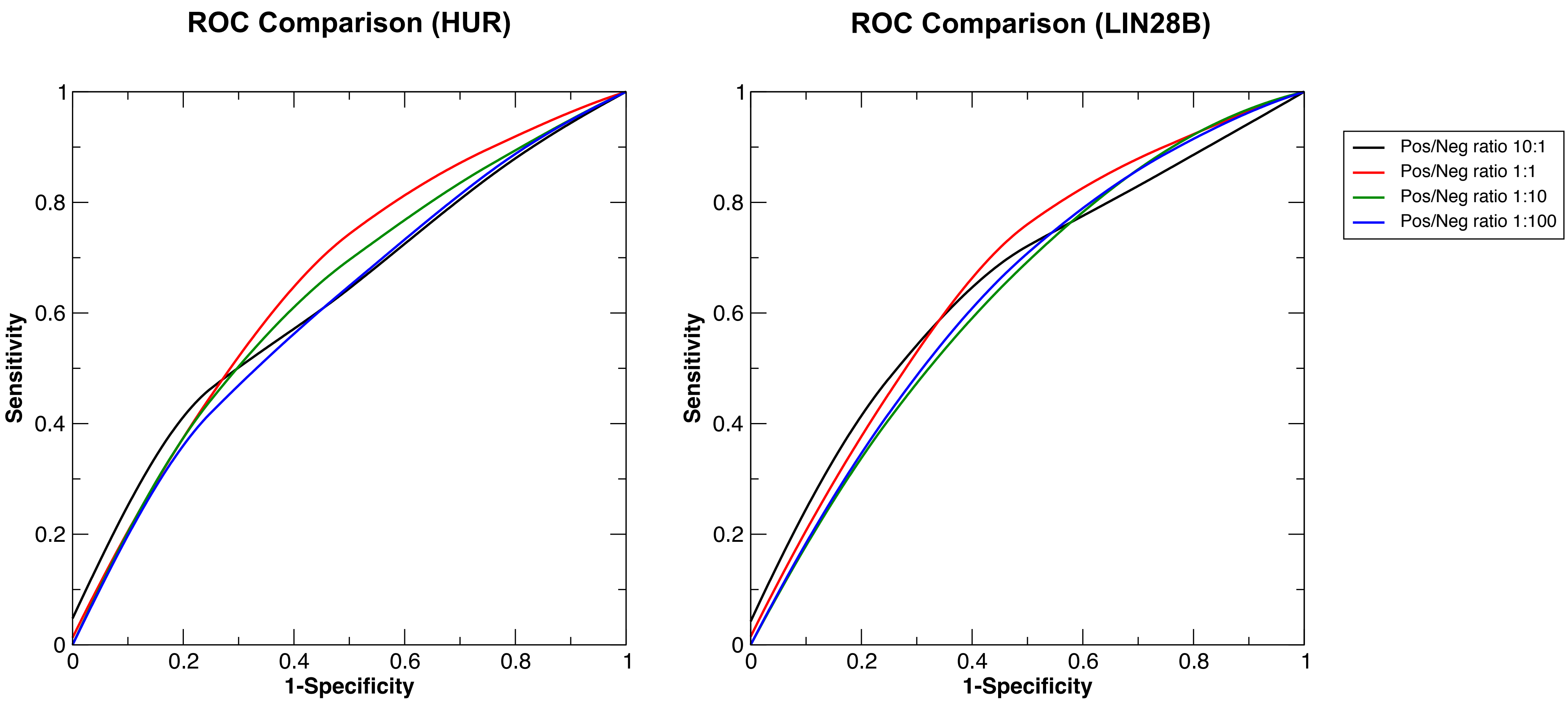
ROC Comparison (HUR)
ROC Comparison (LIN28B)
Sensitivity
1-Specificity
0
0.2
0.4
0.6
0.8
1
Pos/Neg ratio 10:1
Pos/Neg ratio 1:1
Pos/Neg ratio 1:10
Pos/Neg ratio 1:100

Figure S4

B

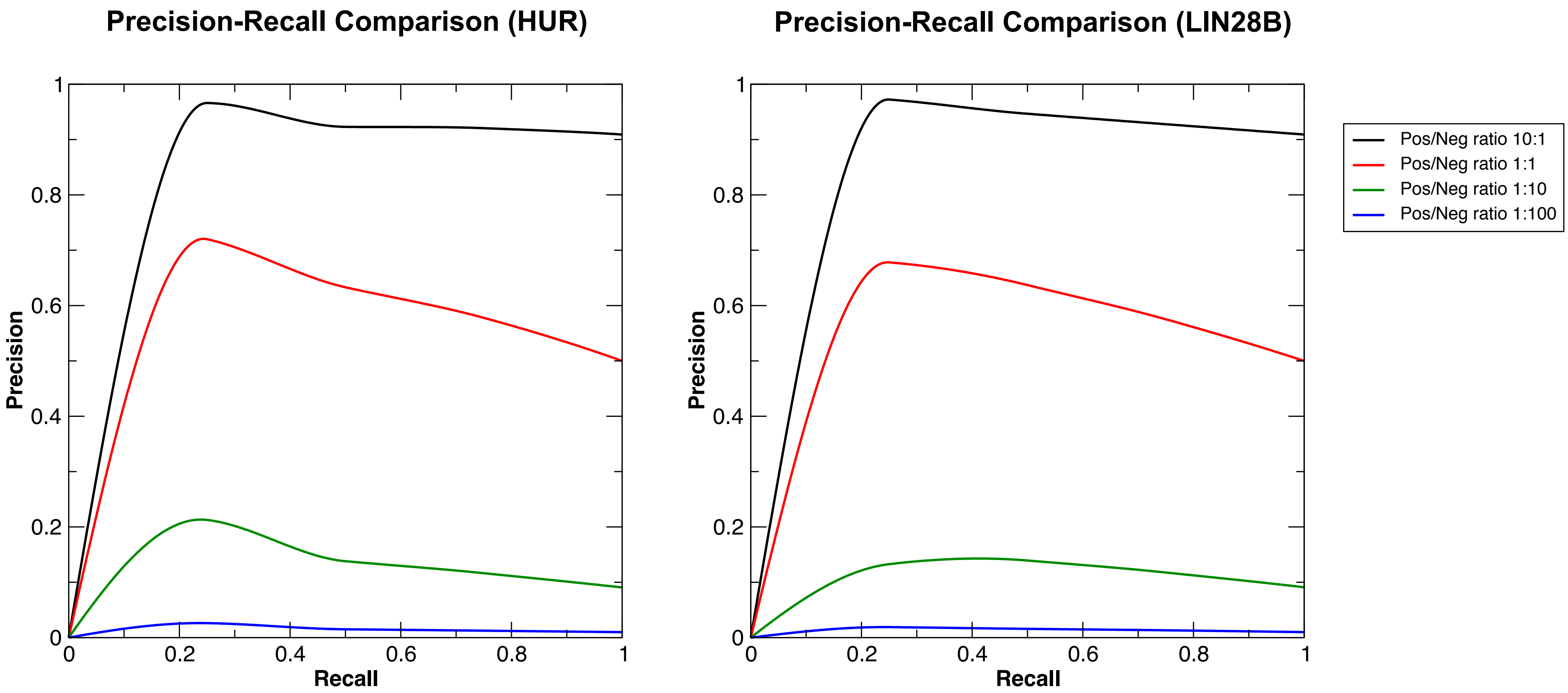
Precision-Recall Comparison (HUR)
Precision-Recall Comparison (LIN28B)
Precision
Recall
0
0.2
0.4
0.6
0.8
1
Pos/Neg ratio 10:1
Pos/Neg ratio 1:1
Pos/Neg ratio 1:10
Pos/Neg ratio 1:100

Figure S4

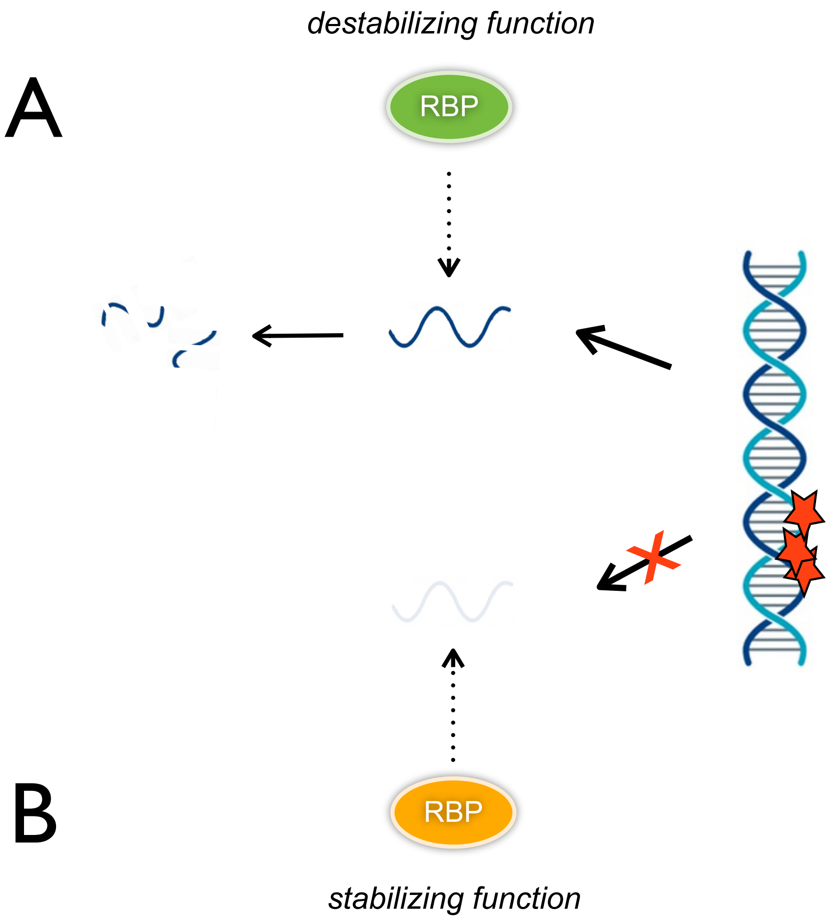

Figure S5

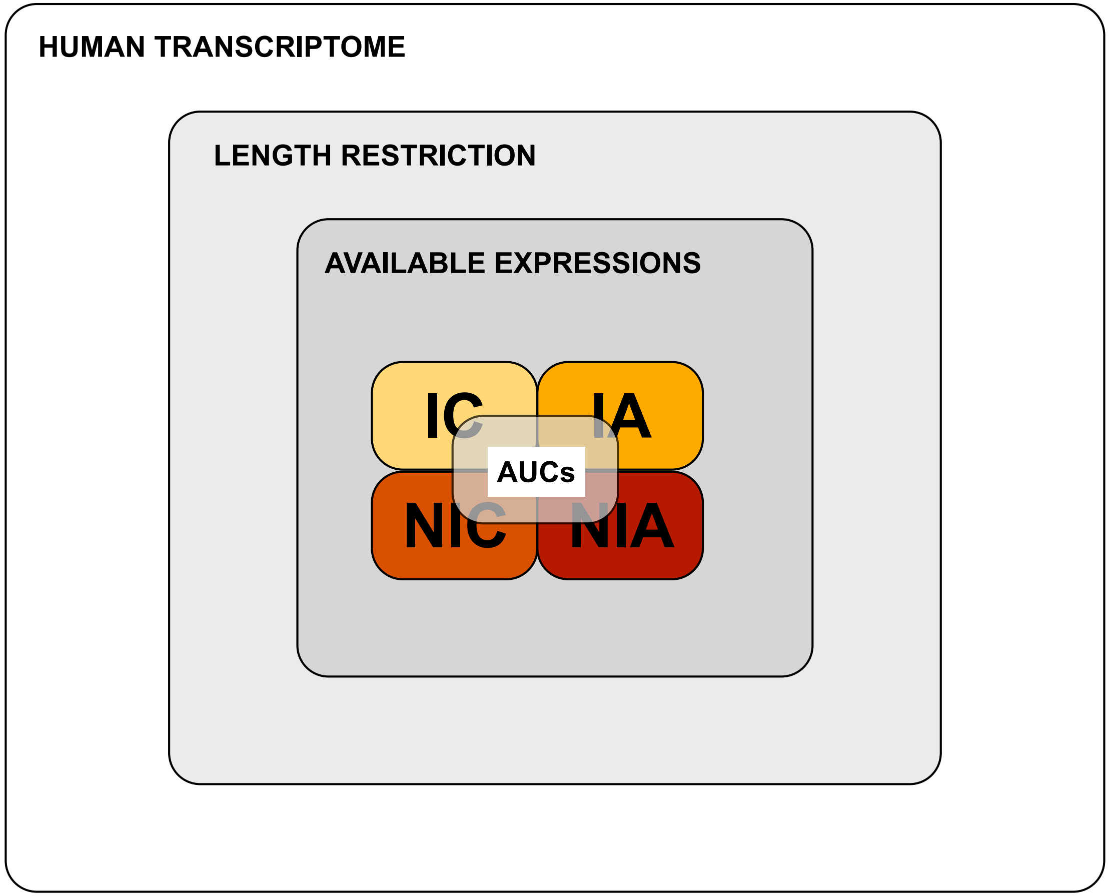
HUMAN TRANSCRIPTOME
LENGTH RESTRICTION
AVAILABLE EXPRESSIONS
IC
IA
AUCs
NIC
NIA

Figure S6

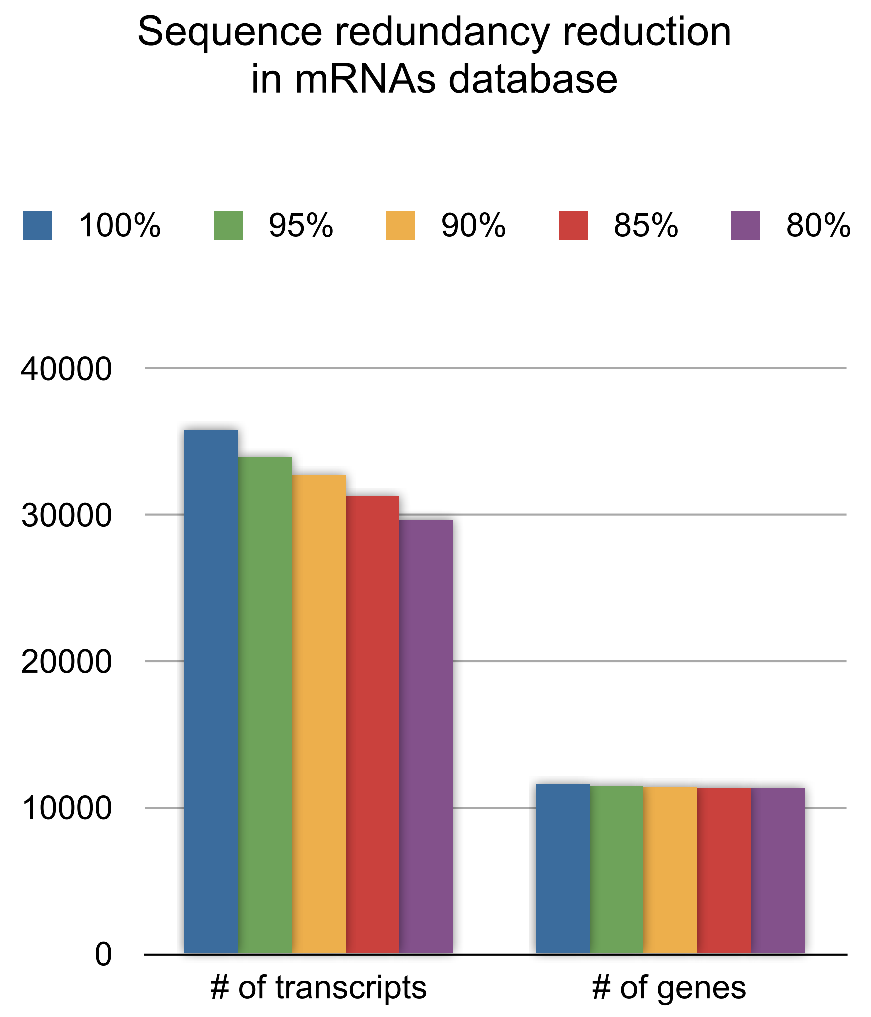
Sequence redundancy reduction
in mRNAs database
100%
95%
90%
85%
80%
40000
30000
20000
10000
0
# of transcripts
# of genes

Figure S7
